# Supplementary material for: Water-Soluble Polyoxometal Clusters of Molybdenum (V) with Pyrazole and Triazole: Synthesis and Study of Cytotoxicity and Antiviral Activity
Source: Molecules. 2023 Dec 14;28(24):8079. doi: 10.3390/molecules28248079 (PMC10745505; doi:10.3390/molecules28248079)

checkCIF/PLATON report

Structure factors have been supplied for datablock(s) jul167-1

THIS REPORT IS FOR GUIDANCE ONLY. IF USED AS PART OF A REVIEW PROCEDURE FOR PUBLICATION, IT SHOULD NOT REPLACE THE EXPERTISE OF AN EXPERIENCED CRYSTALLOGRAPHIC REFEREE.

No syntax errors found. CIF dictionary Interpreting this report

Datablock: jul167-1

Bond precision: C-C = 0.0224 A Wavelength=0.71073

Cell:	a=38.3453(15)	b=38.3453(15)	c=22.8946(11)
	alpha=90	beta=90	gamma=120
Temperature:	150 K		

	Calculated	Reported
Volume	29153(3)	29153(3)
Space group	R 3 c	R 3 c
Hall group	R 3 -2"c	R 3 -2"c
Moiety formula	2(C24 H24 Mo12 N16 O28), 3(C3 H4 N2), 9(O), 8(N)	C24 H24 Mo12 N16 O28, 1.5(C3 H4 N2), 4.5(H2 O), 4(N H4)
Sum formula	C57 H60 Mo24 N46 O65	C28.50 H55 Mo12 N23 O32.50
Mr	4732.07	2391.23
Dx,g cm-3	2.426	2.452
Z	9	18
Mu (mm-1)	2.337	2.338
F000	20268.0	20718.0
F000’	19902.95	
h,k,lmax	59,59,35	58,58,35
Nref	24751[12390]	24695
Tmin,Tmax	0.755,0.792	0.606,0.747
Tmin’	0.755	

Correction method= # Reported T Limits: Tmin=0.606 Tmax=0.747
AbsCorr = MULTI-SCAN

Data completeness= 1.99/1.00 Theta(max)= 33.172

R(reflections)= 0.0514(22592)

wR2(reflections)= 0.1249(24695)

S = 1.069 Npar= 918

The following ALERTS were generated. Each ALERT has the format
test-name_ALERT_alert-type_alert-level.
Click on the hyperlinks for more details of the test.

Alert level A

PLAT430_ALERT_2_A Short Inter D...A Contact O1W ..O3WD . 2.10 Ang.
x,y,z = 1_555 Check

Author Response: This contact corresponds to two disordered water molecules with SOF 0.75 and 0.25 correspondidgly. The other contacts with PLAT430 Alert correspond to the hydrogen bonding with undefined H atoms for ammonia cations and disorderen water molecules.

Alert level B

PLAT043_ALERT_1_B Calculated and Reported Mol. Weight Differ by .. 50.39 Check

Author Response: These difference corresponds to unlocalized H atoms of the water molecules and ammonia cations.

PLAT342_ALERT_3_B Low Bond Precision on C-C Bonds .... 0.02244 Ang.

Author Response: This structure has the best R-factor of 5,14% for the intense reflections. So, some of distances were calculated with rather big uncertanties.

PLAT430_ALERT_2_B Short Inter D...A Contact O1 ..N4N . 2.82 Ang.
x,y,z = 1_555 Check

Author Response: This contact corresponds to two disordered water molecules with SOF 0.75 and 0.25 correspondidgly. The other contacts with PLAT430 Alert correspond to the hydrogen bonding with undefined H atoms for ammonia cations and disorderen water molecules.

PLAT430_ALERT_2_B Short Inter D...A Contact O1W ..O3WA . 2.66 Ang.
x,y,z = 1_555 Check

Author Response: This contact corresponds to two disordered water molecules with SOF 0.75 and 0.25 correspondidgly. The other contacts with PLAT430 Alert correspond to the hydrogen bonding with undefined H atoms for ammonia cations and disorderen water molecules.

```
PLAT430_ALERT_2_B Short Inter D...A Contact  O1W       ..N3N       .       2.67 Ang.
                                                       x,y,z  =      1_555 Check
```

Author Response: This contact corresponds to two disordered water molecules with SOF 0.75 and 0.25 correspondidgly. The other contacts with PLAT430 Alert correspond to the hydrogen bonding with undefined H atoms for ammonia cations and disorderen water molecules.

```
PLAT430_ALERT_2_B Short Inter D...A Contact  O2        ..N3N       .       2.86 Ang.
                                                       x,y,z  =      1_555 Check
```

Author Response: This contact corresponds to two disordered water molecules with SOF 0.75 and 0.25 correspondidgly. The other contacts with PLAT430 Alert correspond to the hydrogen bonding with undefined H atoms for ammonia cations and disorderen water molecules.

```
PLAT430_ALERT_2_B Short Inter D...A Contact  O2WB      ..O10       .       2.80 Ang.
                                 1/3-x+y,2/3-x,-1/3+z  =     15_554 Check
```

Author Response: This contact corresponds to two disordered water molecules with SOF 0.75 and 0.25 correspondidgly. The other contacts with PLAT430 Alert correspond to the hydrogen bonding with undefined H atoms for ammonia cations and disorderen water molecules.

```
PLAT430_ALERT_2_B Short Inter D...A Contact  O2WB      ..O4        .       2.82 Ang.
                                    1/3-y,2/3-x,1/6+z  =     18_554 Check
```

Author Response: This contact corresponds to two disordered water molecules with SOF 0.75 and 0.25 correspondidgly. The other contacts with PLAT430 Alert correspond to the hydrogen bonding with undefined H atoms for ammonia cations and disorderen water molecules.

```
PLAT430_ALERT_2_B Short Inter D...A Contact  O3        ..O5WB      .       2.81 Ang.
                                                       x,y,z  =      1_555 Check
```

Author Response: This contact corresponds to two disordered water molecules with SOF 0.75 and 0.25 correspondidgly. The other contacts with PLAT430 Alert correspond to the hydrogen bonding with undefined H atoms for ammonia cations and disorderen water molecules.

```
PLAT430_ALERT_2_B Short Inter D...A Contact  O3WA      ..N1N       .       2.85 Ang.
                                 1/3-x+y,2/3-x,-1/3+z  =     15_554 Check
```

Author Response: This contact corresponds to two disordered water molecules with SOF 0.75 and 0.25 correspondidgly. The other contacts with PLAT430 Alert correspond to the hydrogen bonding with undefined H atoms for ammonia cations and disorderen water molecules.

```
PLAT430_ALERT_2_B Short Inter D...A Contact  O3WB     ..N2N      .       2.70 Ang.
                                                    x,y,z  =      1_555 Check
```

Author Response: This contact corresponds to two disordered water molecules with SOF 0.75 and 0.25 correspondidgly. The other contacts with PLAT430 Alert correspond to the hydrogen bonding with undefined H atoms for ammonia cations and disorderen water molecules.

```
PLAT430_ALERT_2_B Short Inter D...A Contact  O5       ..N3N      .       2.78 Ang.
                                                -x+y,-x,z  =      3_555 Check
```

Author Response: This contact corresponds to two disordered water molecules with SOF 0.75 and 0.25 correspondidgly. The other contacts with PLAT430 Alert correspond to the hydrogen bonding with undefined H atoms for ammonia cations and disorderen water molecules.

```
PLAT430_ALERT_2_B Short Inter D...A Contact  O7       ..N4N      .       2.84 Ang.
                                             x,x-y,1/2+z  =      5_555 Check
```

Author Response: This contact corresponds to two disordered water molecules with SOF 0.75 and 0.25 correspondidgly. The other contacts with PLAT430 Alert correspond to the hydrogen bonding with undefined H atoms for ammonia cations and disorderen water molecules.

```
PLAT430_ALERT_2_B Short Inter D...A Contact  O4WA     ..N4N      .       2.73 Ang.
                                                    x,y,z  =      1_555 Check
```

Author Response: This contact corresponds to two disordered water molecules with SOF 0.75 and 0.25 correspondidgly. The other contacts with PLAT430 Alert correspond to the hydrogen bonding with undefined H atoms for ammonia cations and disorderen water molecules.

```
PLAT430_ALERT_2_B Short Inter D...A Contact  O4WA     ..O5WB     .       2.75 Ang.
                                            -y,-x,-1/2+z  =      6_554 Check
```

Author Response: This contact corresponds to two disordered water molecules with SOF 0.75 and 0.25 correspondidgly. The other contacts with PLAT430 Alert correspond to the hydrogen bonding with undefined H atoms for ammonia cations and disorderen water molecules.

```
PLAT430_ALERT_2_B Short Inter D...A Contact  O8       ..N2N      .       2.77 Ang.
                                    2/3-y,1/3-x,-1/6+z  =     12_554 Check
```

Author Response: This contact corresponds to two disordered water molecules with SOF 0.75 and 0.25 correspondidgly. The other contacts with PLAT430 Alert correspond to the hydrogen bonding with undefined H atoms for ammonia cations and disorderen water molecules.

```
PLAT430_ALERT_2_B Short Inter D...A Contact  O4WC     ..O5WB     .       2.75 Ang.
                                          -y,-x,-1/2+z  =      6_554 Check
```

Author Response: This contact corresponds to two disordered water molecules with SOF 0.75 and 0.25 correspondidgly. The other contacts with PLAT430 Alert correspond to the hydrogen bonding with undefined H atoms for ammonia cations and disorderen water molecules.

```
PLAT430_ALERT_2_B Short Inter D...A Contact  O12      ..N2N      .       2.87 Ang.
                                  1/3-x+y,-1/3+y,1/6+z  =     16_544 Check
```

Author Response: This contact corresponds to two disordered water molecules with SOF 0.75 and 0.25 correspondidgly. The other contacts with PLAT430 Alert correspond to the hydrogen bonding with undefined H atoms for ammonia cations and disorderen water molecules.

```
PLAT430_ALERT_2_B Short Inter D...A Contact  O26      ..N1N      .       2.86 Ang.
                                                 x,y,z  =      1_555 Check
```

Author Response: This contact corresponds to two disordered water molecules with SOF 0.75 and 0.25 correspondidgly. The other contacts with PLAT430 Alert correspond to the hydrogen bonding with undefined H atoms for ammonia cations and disorderen water molecules.

```
PLAT430_ALERT_2_B Short Inter D...A Contact  O28      ..N2N      .       2.87 Ang.
                                    2/3-y,1/3-x,-1/6+z  =     12_554 Check
```

Author Response: This contact corresponds to two disordered water molecules with SOF 0.75 and 0.25 correspondidgly. The other contacts with PLAT430 Alert correspond to the hydrogen bonding with undefined H atoms for ammonia cations and disorderen water molecules.

```
PLAT430_ALERT_2_B Short Inter D...A Contact  N1N      ..N11S     .       2.86 Ang.
                                     1/3-y,2/3-x,1/6+z  =     18_554 Check
```

Author Response: This contact corresponds to two disordered water molecules with SOF 0.75 and 0.25 correspondidgly. The other contacts with PLAT430 Alert correspond to the hydrogen bonding with undefined H atoms for ammonia cations and disorderen water molecules.

```
PLAT430_ALERT_2_B Short Inter D...A Contact  N4N       ..O4WB     .       2.80 Ang.
                                                      x,y,z  =      1_555 Check
```

Author Response: This contact corresponds to two disordered water molecules with SOF 0.75 and 0.25 correspondidgly. The other contacts with PLAT430 Alert correspond to the hydrogen bonding with undefined H atoms for ammonia cations and disorderen water molecules.

```
PLAT971_ALERT_2_B Check Calcd Resid. Dens.  0.69Ang From Mo12           2.69 eA-3
```

Author Response: This is caused by the presence of large amount of high-electron containig atoms of molibdenum in the small volume of cluster-POM species. It is remarkable that all peaks and holes in the electron difference maps are not exceed the 1/10 of Mo.

Alert level C

```
PLAT041_ALERT_1_C Calc. and Reported SumFormula    Strings  Differ     Please Check
PLAT042_ALERT_1_C Calc. and Reported MoietyFormula Strings  Differ     Please Check
PLAT068_ALERT_1_C Reported F000 Differs from Calcd (or Missing)...     Please Check
PLAT214_ALERT_2_C Atom O3WC      (Anion/Solvent) ADP max/min Ratio        4.9 oblate
PLAT214_ALERT_2_C Atom O3WD      (Anion/Solvent) ADP max/min Ratio        4.9 oblate
PLAT243_ALERT_4_C High   'Solvent' Ueq as Compared to Neighbors of       C13S Check
PLAT243_ALERT_4_C High   'Solvent' Ueq as Compared to Neighbors of       C15S Check
PLAT244_ALERT_4_C Low    'Solvent' Ueq as Compared to Neighbors of       N11S Check
PLAT420_ALERT_2_C D-H Bond Without Acceptor  N21S     --H21S     .     Please Check
PLAT430_ALERT_2_C Short Inter D...A Contact  O2WB      ..O9       .       2.86 Ang.
                                                   -y,x-y,z  =      2_555 Check
```

Author Response: This contact corresponds to two disordered water molecules with SOF 0.75 and 0.25 correspondidgly. The other contacts with PLAT430 Alert correspond to the hydrogen bonding with undefined H atoms for ammonia cations and disorderen water molecules.

```
PLAT430_ALERT_2_C Short Inter D...A Contact  O3        ..O5WA     .       2.88 Ang.
                                                      x,y,z  =      1_555 Check
```

Author Response: This contact corresponds to two disordered water molecules with SOF 0.75 and 0.25 correspondidgly. The other contacts with PLAT430 Alert correspond to the hydrogen bonding with undefined H atoms for ammonia cations and disorderen water molecules.

```
PLAT430_ALERT_2_C Short Inter D...A Contact  O6       ..N1N      .       2.89 Ang.
                                                    x,y,z  =      1_555 Check
```

Author Response: This contact corresponds to two disordered water molecules with SOF 0.75 and 0.25 correspondidgly. The other contacts with PLAT430 Alert correspond to the hydrogen bonding with undefined H atoms for ammonia cations and disorderen water molecules.

```
PLAT430_ALERT_2_C Short Inter D...A Contact  O11      ..N1N      .       2.88 Ang.
                                 1/3-x+y,-1/3+y,1/6+z  =     16_544 Check
```

Author Response: This contact corresponds to two disordered water molecules with SOF 0.75 and 0.25 correspondidgly. The other contacts with PLAT430 Alert correspond to the hydrogen bonding with undefined H atoms for ammonia cations and disorderen water molecules.

```
PLAT430_ALERT_2_C Short Inter D...A Contact  O27      ..N4N      .       2.88 Ang.
                                              x,x-y,1/2+z  =      5_555 Check
```

Author Response: This contact corresponds to two disordered water molecules with SOF 0.75 and 0.25 correspondidgly. The other contacts with PLAT430 Alert correspond to the hydrogen bonding with undefined H atoms for ammonia cations and disorderen water molecules.

```
PLAT911_ALERT_3_C Missing FCF Refl Between Thmin & STh/L=    0.600          4 Report
PLAT971_ALERT_2_C Check Calcd Resid. Dens.  0.86Ang From Mo8            2.24 eA-3
```

Author Response: This is caused by the presence of large amount of high-electron containig atoms of molibdenum in the small volume of cluster-POM species. It is remarkable that all peaks and holes in the electron difference maps are not exceed the 1/10 of Mo.

```
PLAT971_ALERT_2_C Check Calcd Resid. Dens.  0.67Ang From Mo9            2.17 eA-3
```

Author Response: This is caused by the presence of large amount of high-electron containig atoms of molibdenum in the small volume of cluster-POM species. It is remarkable that all peaks and holes in the electron difference maps are not exceed the 1/10 of Mo.

```
PLAT971_ALERT_2_C Check Calcd Resid. Dens.  0.79Ang From Mo4            2.03 eA-3
```

Author Response: This is caused by the presence of large amount of high-electron containig atoms of molibdenum in the small volume of cluster-POM species. It is remarkable that all peaks and holes in the electron difference maps are not exceed the 1/10 of Mo.

```
PLAT971_ALERT_2_C Check Calcd Resid. Dens.  0.85Ang From Mo6            1.99 eA-3
```

Author Response: This is caused by the presence of large amount of high-electron containig atoms of molibdenum in the small volume of cluster-POM species. It is remarkable that all peaks and holes in the electron difference maps are not exceed the 1/10 of Mo.

```
PLAT971_ALERT_2_C Check Calcd Resid. Dens.  0.75Ang From Mo11           1.95 eA-3
```

Author Response: This is caused by the presence of large amount of high-electron containig atoms of molibdenum in the small volume of cluster-POM species. It is remarkable that all peaks and holes in the electron difference maps are not exceed the 1/10 of Mo.

```
PLAT971_ALERT_2_C Check Calcd Resid. Dens.  0.85Ang From Mo2            1.83 eA-3
```

Author Response: This is caused by the presence of large amount of high-electron containig atoms of molibdenum in the small volume of cluster-POM species. It is remarkable that all peaks and holes in the electron difference maps are not exceed the 1/10 of Mo.

```
PLAT971_ALERT_2_C Check Calcd Resid. Dens.  0.73Ang From Mo12           1.83 eA-3
```

Author Response: This is caused by the presence of large amount of high-electron containig atoms of molibdenum in the small volume of cluster-POM species. It is remarkable that all peaks and holes in the electron difference maps are not exceed the 1/10 of Mo.

```
PLAT971_ALERT_2_C Check Calcd Resid. Dens.  0.87Ang From Mo4            1.80 eA-3
```

Author Response: This is caused by the presence of large amount of high-electron containig atoms of molibdenum in the small volume of cluster-POM species. It is remarkable that all peaks and holes in the electron difference maps are not exceed the 1/10 of Mo.

```
PLAT971_ALERT_2_C Check Calcd Resid. Dens.  0.84Ang From Mo5            1.79 eA-3
```

Author Response: This is caused by the presence of large amount of high-electron containig atoms of molibdenum in the small volume of cluster-POM species. It is remarkable that all peaks and holes in the electron difference maps are not exceed the 1/10 of Mo.

PLAT971_ALERT_2_C Check Calcd Resid. Dens. 0.89Ang From Mo8   1.75 eA-3

Author Response: This is caused by the presence of large amount of high-electron containig atoms of molibdenum in the small volume of cluster-POM species. It is remarkable that all peaks and holes in the electron difference maps are not exceed the 1/10 of Mo.

PLAT971_ALERT_2_C Check Calcd Resid. Dens. 0.68Ang From Mo10   1.74 eA-3

Author Response: This is caused by the presence of large amount of high-electron containig atoms of molibdenum in the small volume of cluster-POM species. It is remarkable that all peaks and holes in the electron difference maps are not exceed the 1/10 of Mo.

PLAT971_ALERT_2_C Check Calcd Resid. Dens. 0.88Ang From Mo5   1.73 eA-3

Author Response: This is caused by the presence of large amount of high-electron containig atoms of molibdenum in the small volume of cluster-POM species. It is remarkable that all peaks and holes in the electron difference maps are not exceed the 1/10 of Mo.

PLAT971_ALERT_2_C Check Calcd Resid. Dens. 0.86Ang From Mo9   1.68 eA-3

Author Response: This is caused by the presence of large amount of high-electron containig atoms of molibdenum in the small volume of cluster-POM species. It is remarkable that all peaks and holes in the electron difference maps are not exceed the 1/10 of Mo.

PLAT971_ALERT_2_C Check Calcd Resid. Dens. 0.75Ang From Mo11   1.66 eA-3

Author Response: This is caused by the presence of large amount of high-electron containig atoms of molibdenum in the small volume of cluster-POM species. It is remarkable that all peaks and holes in the electron difference maps are not exceed the 1/10 of Mo.

PLAT971_ALERT_2_C Check Calcd Resid. Dens. 0.87Ang From Mo1   1.63 eA-3

Author Response: This is caused by the presence of large amount of high-electron containig atoms of molibdenum in the small volume of cluster-POM species. It is remarkable that all peaks and holes in the electron difference maps are not exceed the 1/10 of Mo.

PLAT971_ALERT_2_C Check Calcd Resid. Dens. 0.84Ang From Mo5 1.62 eA-3

Author Response: This is caused by the presence of large amount of high-electron containig atoms of molibdenum in the small volume of cluster-POM species. It is remarkable that all peaks and holes in the electron difference maps are not exceed the 1/10 of Mo.

PLAT971_ALERT_2_C Check Calcd Resid. Dens. 0.87Ang From Mo8 1.60 eA-3

Author Response: This is caused by the presence of large amount of high-electron containig atoms of molibdenum in the small volume of cluster-POM species. It is remarkable that all peaks and holes in the electron difference maps are not exceed the 1/10 of Mo.

PLAT971_ALERT_2_C Check Calcd Resid. Dens. 0.83Ang From Mo7 1.55 eA-3

Author Response: This is caused by the presence of large amount of high-electron containig atoms of molibdenum in the small volume of cluster-POM species. It is remarkable that all peaks and holes in the electron difference maps are not exceed the 1/10 of Mo.

PLAT971_ALERT_2_C Check Calcd Resid. Dens. 0.86Ang From Mo1 1.54 eA-3

Author Response: This is caused by the presence of large amount of high-electron containig atoms of molibdenum in the small volume of cluster-POM species. It is remarkable that all peaks and holes in the electron difference maps are not exceed the 1/10 of Mo.

PLAT971_ALERT_2_C Check Calcd Resid. Dens. 0.97Ang From Mo6 1.54 eA-3

Author Response: This is caused by the presence of large amount of high-electron containig atoms of molibdenum in the small volume of cluster-POM species. It is remarkable that all peaks and holes in the electron difference maps are not exceed the 1/10 of Mo.

PLAT971_ALERT_2_C Check Calcd Resid. Dens. 0.86Ang From Mo7 1.52 eA-3

Author Response: This is caused by the presence of large amount of high-electron containig atoms of molibdenum in the small volume of cluster-POM species. It is remarkable that all peaks and holes in the electron difference maps are not exceed the 1/10 of Mo.

```
PLAT971_ALERT_2_C Check Calcd Resid. Dens.  0.73Ang From Mo10           1.52 eA-3
```

Author Response: This is caused by the presence of large amount of high-electron containig atoms of molibdenum in the small volume of cluster-POM species. It is remarkable that all peaks and holes in the electron difference maps are not exceed the 1/10 of Mo.

```
PLAT971_ALERT_2_C Check Calcd Resid. Dens.  0.78Ang From O2             1.51 eA-3
```

Author Response: This is caused by the presence of large amount of high-electron containig atoms of molibdenum in the small volume of cluster-POM species. It is remarkable that all peaks and holes in the electron difference maps are not exceed the 1/10 of Mo.

```
PLAT972_ALERT_2_C Check Calcd Resid. Dens.  0.58Ang From Mo12          -2.06 eA-3
PLAT972_ALERT_2_C Check Calcd Resid. Dens.  0.60Ang From Mo9           -1.77 eA-3
PLAT972_ALERT_2_C Check Calcd Resid. Dens.  0.66Ang From Mo11          -1.63 eA-3
PLAT972_ALERT_2_C Check Calcd Resid. Dens.  0.63Ang From Mo11          -1.63 eA-3
PLAT976_ALERT_2_C Check Calcd Resid. Dens.  0.89Ang From O13     .     -1.03 eA-3
PLAT977_ALERT_2_C Check Negative Difference Density on H24S      .     -0.33 eA-3
PLAT977_ALERT_2_C Check Negative Difference Density on H25S      .     -0.38 eA-3
PLAT977_ALERT_2_C Check Negative Difference Density on H75       .     -0.34 eA-3
```

Alert level G

```
FORMU01_ALERT_2_G  There is a discrepancy between the atom counts in the
            _chemical_formula_sum and the formula from the _atom_site* data.
            Atom count from _chemical_formula_sum:C28.5 H55 Mo12 N23 O32.5
            Atom count from the _atom_site data:  C28.5 H30 Mo12 N23 O32.5
CELLZ01_ALERT_1_G Difference between formula and atom_site contents detected.
CELLZ01_ALERT_1_G WARNING: H atoms missing from atom site list. Is this intentional?
           From the CIF: _cell_formula_units_Z   18
           From the CIF: _chemical_formula_sum  C28.50 H55 Mo12 N23 O32.50
           TEST: Compare cell contents of formula and atom_site data

           atom    Z*formula  cif sites diff
           C        513.00    513.00    0.00
           H        990.00    540.00  450.00
           Mo       216.00    216.00    0.00
           N        414.00    414.00    0.00
           O        585.00    585.00    0.00
PLAT002_ALERT_2_G Number of Distance or Angle Restraints on AtSite          9 Note
PLAT003_ALERT_2_G Number of Uiso or Uij Restrained non-H Atoms ...          2 Report
PLAT007_ALERT_5_G Number of Unrefined Donor-H Atoms .............          2 Report
PLAT045_ALERT_1_G Calculated and Reported Z Differ by a Factor ...      0.500 Check
PLAT083_ALERT_2_G SHELXL Second Parameter in WGHT  Unusually Large     637.57 Why ?
PLAT171_ALERT_4_G The CIF-Embedded .res File Contains EADP Records          5 Report
PLAT172_ALERT_4_G The CIF-Embedded .res File Contains DFIX Records          6 Report
PLAT186_ALERT_4_G The CIF-Embedded .res File Contains ISOR Records          1 Report
PLAT300_ALERT_4_G Atom Site Occupancy of N21S       Constrained at        0.5 Check
PLAT300_ALERT_4_G Atom Site Occupancy of N22S       Constrained at        0.5 Check
PLAT300_ALERT_4_G Atom Site Occupancy of C23S       Constrained at        0.5 Check
PLAT300_ALERT_4_G Atom Site Occupancy of C24S       Constrained at        0.5 Check
```

```
PLAT300_ALERT_4_G Atom Site Occupancy of C25S       Constrained at        0.5 Check
PLAT300_ALERT_4_G Atom Site Occupancy of H21S       Constrained at        0.5 Check
PLAT300_ALERT_4_G Atom Site Occupancy of H23S       Constrained at        0.5 Check
PLAT300_ALERT_4_G Atom Site Occupancy of H24S       Constrained at        0.5 Check
PLAT300_ALERT_4_G Atom Site Occupancy of H25S       Constrained at        0.5 Check
PLAT300_ALERT_4_G Atom Site Occupancy of O1W        Constrained at       0.75 Check
PLAT300_ALERT_4_G Atom Site Occupancy of O3WA       Constrained at        0.5 Check
PLAT300_ALERT_4_G Atom Site Occupancy of O3WB       Constrained at        0.5 Check
PLAT300_ALERT_4_G Atom Site Occupancy of O4WA       Constrained at        0.5 Check
PLAT300_ALERT_4_G Atom Site Occupancy of O4WC       Constrained at        0.5 Check
PLAT300_ALERT_4_G Atom Site Occupancy of O3WC       Constrained at       0.25 Check
PLAT300_ALERT_4_G Atom Site Occupancy of O3WD       Constrained at       0.25 Check
PLAT300_ALERT_4_G Atom Site Occupancy of O4WB       Constrained at       0.25 Check
PLAT302_ALERT_4_G Anion/Solvent/Minor-Residue Disorder (Resd  3  )       100% Note
PLAT302_ALERT_4_G Anion/Solvent/Minor-Residue Disorder (Resd  4  )       100% Note
PLAT302_ALERT_4_G Anion/Solvent/Minor-Residue Disorder (Resd  5  )       100% Note
PLAT302_ALERT_4_G Anion/Solvent/Minor-Residue Disorder (Resd  6  )       100% Note
PLAT302_ALERT_4_G Anion/Solvent/Minor-Residue Disorder (Resd  7  )       100% Note
PLAT302_ALERT_4_G Anion/Solvent/Minor-Residue Disorder (Resd  8  )       100% Note
PLAT302_ALERT_4_G Anion/Solvent/Minor-Residue Disorder (Resd  9  )       100% Note
PLAT302_ALERT_4_G Anion/Solvent/Minor-Residue Disorder (Resd 10  )       100% Note
PLAT302_ALERT_4_G Anion/Solvent/Minor-Residue Disorder (Resd 15  )       100% Note
PLAT302_ALERT_4_G Anion/Solvent/Minor-Residue Disorder (Resd 16  )       100% Note
PLAT302_ALERT_4_G Anion/Solvent/Minor-Residue Disorder (Resd 17  )       100% Note
PLAT302_ALERT_4_G Anion/Solvent/Minor-Residue Disorder (Resd 18  )       100% Note
PLAT302_ALERT_4_G Anion/Solvent/Minor-Residue Disorder (Resd 19  )       100% Note
PLAT304_ALERT_4_G Non-Integer Number of Atoms in ..... (Resd  3  )       4.50 Check
PLAT304_ALERT_4_G Non-Integer Number of Atoms in ..... (Resd  4  )       0.75 Check
PLAT304_ALERT_4_G Non-Integer Number of Atoms in ..... (Resd  5  )       0.56 Check
PLAT304_ALERT_4_G Non-Integer Number of Atoms in ..... (Resd  6  )       0.50 Check
PLAT304_ALERT_4_G Non-Integer Number of Atoms in ..... (Resd  7  )       0.50 Check
PLAT304_ALERT_4_G Non-Integer Number of Atoms in ..... (Resd  8  )       0.50 Check
PLAT304_ALERT_4_G Non-Integer Number of Atoms in ..... (Resd  9  )       0.17 Check
PLAT304_ALERT_4_G Non-Integer Number of Atoms in ..... (Resd 10  )       0.22 Check
PLAT304_ALERT_4_G Non-Integer Number of Atoms in ..... (Resd 15  )       0.44 Check
PLAT304_ALERT_4_G Non-Integer Number of Atoms in ..... (Resd 16  )       0.25 Check
PLAT304_ALERT_4_G Non-Integer Number of Atoms in ..... (Resd 17  )       0.25 Check
PLAT304_ALERT_4_G Non-Integer Number of Atoms in ..... (Resd 18  )       0.25 Check
PLAT304_ALERT_4_G Non-Integer Number of Atoms in ..... (Resd 19  )       0.11 Check
PLAT311_ALERT_2_G Isolated Disordered Oxygen Atom (No H's ?) .....        O1W Check
PLAT311_ALERT_2_G Isolated Disordered Oxygen Atom (No H's ?) .....       O2WB Check
PLAT311_ALERT_2_G Isolated Disordered Oxygen Atom (No H's ?) .....       O3WA Check
PLAT311_ALERT_2_G Isolated Disordered Oxygen Atom (No H's ?) .....       O3WB Check
PLAT311_ALERT_2_G Isolated Disordered Oxygen Atom (No H's ?) .....       O4WA Check
PLAT311_ALERT_2_G Isolated Disordered Oxygen Atom (No H's ?) .....       O4WC Check
PLAT311_ALERT_2_G Isolated Disordered Oxygen Atom (No H's ?) .....       O5WA Check
PLAT311_ALERT_2_G Isolated Disordered Oxygen Atom (No H's ?) .....       O2WA Check
PLAT311_ALERT_2_G Isolated Disordered Oxygen Atom (No H's ?) .....       O3WC Check
PLAT311_ALERT_2_G Isolated Disordered Oxygen Atom (No H's ?) .....       O3WD Check
PLAT311_ALERT_2_G Isolated Disordered Oxygen Atom (No H's ?) .....       O4WB Check
PLAT311_ALERT_2_G Isolated Disordered Oxygen Atom (No H's ?) .....       O5WB Check
PLAT432_ALERT_2_G Short Inter X...Y Contact  O7       ..C23S     .       2.97 Ang.
                                    1/3-x+y,-1/3+y,1/6+z  =     16_544 Check
PLAT432_ALERT_2_G Short Inter X...Y Contact  C13S     ..C24S     .       2.98 Ang.
                                                 x,y,z  =      1_555 Check
PLAT432_ALERT_2_G Short Inter X...Y Contact  C13S     ..C25S     .       3.12 Ang.
                                                 x,y,z  =      1_555 Check
```

```
PLAT432_ALERT_2_G Short Inter X...Y Contact  C14S      ..C24S     .       3.17 Ang.
                                                      x,y,z  =      1_555 Check
PLAT720_ALERT_4_G Number of Unusual/Non-Standard Labels .........         11 Note
PLAT860_ALERT_3_G Number of Least-Squares Restraints ............         19 Note
PLAT883_ALERT_1_G No Info/Value for _atom_sites_solution_primary .     Please Do !
PLAT910_ALERT_3_G Missing # of FCF Reflection(s) Below Theta(Min).          2 Note
PLAT912_ALERT_4_G Missing # of FCF Reflections Above STh/L=  0.600          6 Note
PLAT933_ALERT_2_G Number of HKL-OMIT Records in Embedded .res File          7 Note
PLAT961_ALERT_5_G Dataset Contains no Negative Intensities .......     Please Check
PLAT965_ALERT_2_G The SHELXL WEIGHT Optimisation has not Converged     Please Check
PLAT978_ALERT_2_G Number C-C Bonds with Positive Residual Density.          0 Info
```

```
  1 ALERT level A = Most likely a serious problem - resolve or explain
 23 ALERT level B = A potentially serious problem, consider carefully
 46 ALERT level C = Check. Ensure it is not caused by an omission or oversight
 79 ALERT level G = General information/check it is not something unexpected

  8 ALERT type 1 CIF construction/syntax error, inconsistent or missing data
 84 ALERT type 2 Indicator that the structure model may be wrong or deficient
  4 ALERT type 3 Indicator that the structure quality may be low
 51 ALERT type 4 Improvement, methodology, query or suggestion
  2 ALERT type 5 Informative message, check
```

It is advisable to attempt to resolve as many as possible of the alerts in all categories. Often the minor alerts point to easily fixed oversights, errors and omissions in your CIF or refinement strategy, so attention to these fine details can be worthwhile. In order to resolve some of the more serious problems it may be necessary to carry out additional measurements or structure refinements. However, the purpose of your study may justify the reported deviations and the more serious of these should normally be commented upon in the discussion or experimental section of a paper or in the "special_details" fields of the CIF. checkCIF was carefully designed to identify outliers and unusual parameters, but every test has its limitations and alerts that are not important in a particular case may appear. Conversely, the absence of alerts does not guarantee there are no aspects of the results needing attention. It is up to the individual to critically assess their own results and, if necessary, seek expert advice.

Publication of your CIF in IUCr journals

A basic structural check has been run on your CIF. These basic checks will be run on all CIFs submitted for publication in IUCr journals (*Acta Crystallographica*, *Journal of Applied Crystallography*, *Journal of Synchrotron Radiation*); however, if you intend to submit to *Acta Crystallographica Section C* or *E* or *IUCrData*, you should make sure that full publication checks are run on the final version of your CIF prior to submission.

Publication of your CIF in other journals

Please refer to the *Notes for Authors* of the relevant journal for any special instructions relating to CIF submission.

PLATON version of 06/07/2023; check.def file version of 30/06/2023

Datablock jul167-1 - ellipsoid plot

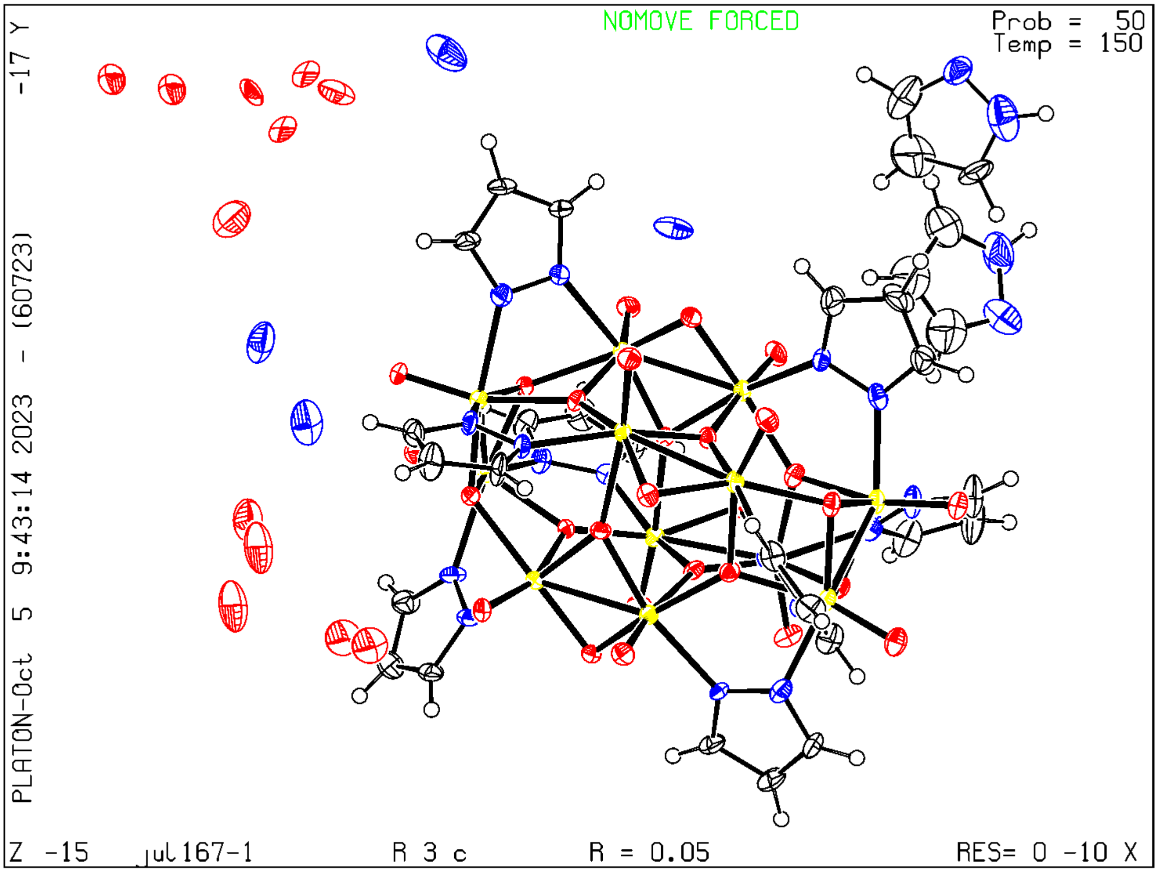

Supplement: Supplementary file 1 [file molecules-28-08079-s001.zip › Compound 1 checkcif.pdf]
