# Supplementary material for: Water-Soluble Polyoxometal Clusters of Molybdenum (V) with Pyrazole and Triazole: Synthesis and Study of Cytotoxicity and Antiviral Activity
Source: Molecules. 2023 Dec 14;28(24):8079. doi: 10.3390/molecules28248079 (PMC10745505; doi:10.3390/molecules28248079)

checkCIF/PLATON report

Structure factors have been supplied for datablock(s) jul167-4

THIS REPORT IS FOR GUIDANCE ONLY. IF USED AS PART OF A REVIEW PROCEDURE FOR PUBLICATION, IT SHOULD NOT REPLACE THE EXPERTISE OF AN EXPERIENCED CRYSTALLOGRAPHIC REFEREE.

No syntax errors found. CIF dictionary Interpreting this report

Datablock: jul167-4

```
Bond precision:   N- C = 0.0081 A          Wavelength=0.71073

Cell:              a=26.6106(4)      b=26.6106(4)      c=26.6106(4)
                   alpha=90          beta=90           gamma=90
Temperature:       150 K
```

	Calculated	Reported
Volume	18843.6(8)	18843.6(8)
Space group	I -4 3 d	I -4 3 d
Hall group	I -4bd 2c 3	I -4bd 2c 3
Moiety formula	12(C16 H16 Mo12 N24 O28), 3(O4), 4(O3), 6(O2), 84(O), 48(N)	4(N H4), C16 H16 Mo12 N24 O28, 10(H2 O)
Sum formula	C192 H192 Mo144 N336 O456	C16 H52 Mo12 N28 O38
Mr	28318.21	2396.13
Dx,g cm-3	2.496	2.534
Z	1	12
Mu (mm-1)	2.419	2.420
F000	13392.0	13824.0
F000’	13148.99	
h,k,lmax	38,38,38	37,38,38
Nref	4795[2538]	4789
Tmin,Tmax	0.865,0.865	0.684,0.746
Tmin’	0.865	

```
Correction method= # Reported T Limits: Tmin=0.684 Tmax=0.746
AbsCorr = MULTI-SCAN

Data completeness= 1.89/1.00        Theta(max)= 30.495
```

R(reflections)= 0.0243(4537) wR2(reflections)= 0.0565(4789)

S = 1.131 Npar= 254

The following ALERTS were generated. Each ALERT has the format
test-name_ALERT_alert-type_alert-level.
Click on the hyperlinks for more details of the test.

Alert level B

PLAT043_ALERT_1_B Calculated and Reported Mol. Weight Differ by .. 435.35 Check

Author Response: Reported formula contain 36 unlocalized H atoms of 10 disordered solvate water molecules and 4 ammonia cations. Also the checking algorithm calculate the number of the formula units as 1 instead the real 12.

PLAT430_ALERT_2_B Short Inter D...A Contact O5W ..N1N . 2.85 Ang.
z,x,y = 11_555 Check

Author Response: The H atoms of the disordered solvate water molecules are not localized. This contact corresponds to the H bond.

PLAT430_ALERT_2_B Short Inter D...A Contact N1N ..N14 . 2.87 Ang.
-1/4+y,-1/4+x,-1/4+z = 44_444 Check

Author Response: The H atoms of the disordered solvate water molecules are not localized. This contact corresponds to the H bond.

Alert level C

PLAT041_ALERT_1_C Calc. and Reported SumFormula Strings Differ Please Check
PLAT042_ALERT_1_C Calc. and Reported MoietyFormula Strings Differ Please Check
PLAT068_ALERT_1_C Reported F000 Differs from Calcd (or Missing)... Please Check
PLAT241_ALERT_2_C High 'MainMol' Ueq as Compared to Neighbors of N14 Check
PLAT250_ALERT_2_C Large U3/U1 Ratio for Average U(i,j) Tensor .... 2.1 Note
PLAT430_ALERT_2_C Short Inter D...A Contact O3 ..N1N . 2.88 Ang.
x,y,z = 1_555 Check

Author Response: The H atoms of the disordered solvate water molecules are not localized. This contact corresponds to the H bond.

PLAT911_ALERT_3_C Missing FCF Refl Between Thmin & STh/L= 0.600 3 Report
PLAT975_ALERT_2_C Check Calcd Resid. Dens. 0.72Ang From N1N . 0.70 eA-3
PLAT975_ALERT_2_C Check Calcd Resid. Dens. 0.91Ang From N1N . 0.47 eA-3
PLAT975_ALERT_2_C Check Calcd Resid. Dens. 1.00Ang From N1N . 0.46 eA-3
PLAT975_ALERT_2_C Check Calcd Resid. Dens. 1.07Ang From O2 . 0.45 eA-3
PLAT975_ALERT_2_C Check Calcd Resid. Dens. 1.07Ang From N1N . 0.42 eA-3

Alert level G

```
FORMU01_ALERT_2_G  There is a discrepancy between the atom counts in the
            _chemical_formula_sum and the formula from the _atom_site* data.
            Atom count from _chemical_formula_sum:C16 H52 Mo12 N28 O38
            Atom count from the _atom_site data:  C16 H16 Mo12 N28 O38.
CELLZ01_ALERT_1_G Difference between formula and atom_site contents detected.
CELLZ01_ALERT_1_G WARNING: H atoms missing from atom site list. Is this intentional?
           From the CIF: _cell_formula_units_Z   12
           From the CIF: _chemical_formula_sum  C16 H52 Mo12 N28 O38
           TEST: Compare cell contents of formula and atom_site data

           atom    Z*formula  cif sites diff
           C         192.00    192.00    0.00
           H         624.00    192.00  432.00
           Mo        144.00    144.00    0.00
           N         336.00    336.00    0.00
           O         456.00    456.00   -0.00
PLAT003_ALERT_2_G Number of Uiso or Uij Restrained non-H Atoms ...          4 Report
PLAT045_ALERT_1_G Calculated and Reported Z Differ by a Factor ...      0.083 Check
PLAT083_ALERT_2_G SHELXL Second Parameter in WGHT  Unusually Large      64.25 Why ?
PLAT171_ALERT_4_G The CIF-Embedded .res File Contains EADP Records          1 Report
PLAT186_ALERT_4_G The CIF-Embedded .res File Contains ISOR Records          1 Report
PLAT300_ALERT_4_G Atom Site Occupancy of O3W       Constrained at       0.25 Check
PLAT300_ALERT_4_G Atom Site Occupancy of O1W       Constrained at       0.25 Check
PLAT300_ALERT_4_G Atom Site Occupancy of O7W       Constrained at       0.25 Check
PLAT300_ALERT_4_G Atom Site Occupancy of O2W       Constrained at       0.75 Check
PLAT300_ALERT_4_G Atom Site Occupancy of O5W       Constrained at        0.5 Check
PLAT300_ALERT_4_G Atom Site Occupancy of O6W       Constrained at       0.75 Check
PLAT300_ALERT_4_G Atom Site Occupancy of O4W       Constrained at      0.375 Check
PLAT300_ALERT_4_G Atom Site Occupancy of O8W       Constrained at     0.1667 Check
PLAT300_ALERT_4_G Atom Site Occupancy of O9W       Constrained at       0.25 Check
PLAT300_ALERT_4_G Atom Site Occupancy of O11W      Constrained at      0.375 Check
PLAT300_ALERT_4_G Atom Site Occupancy of O12W      Constrained at       0.25 Check
PLAT302_ALERT_4_G Anion/Solvent/Minor-Residue Disorder (Resd  2  )       100% Note
PLAT302_ALERT_4_G Anion/Solvent/Minor-Residue Disorder (Resd  3  )       100% Note
PLAT302_ALERT_4_G Anion/Solvent/Minor-Residue Disorder (Resd  4  )       100% Note
PLAT302_ALERT_4_G Anion/Solvent/Minor-Residue Disorder (Resd  5  )       100% Note
PLAT302_ALERT_4_G Anion/Solvent/Minor-Residue Disorder (Resd  6  )       100% Note
PLAT302_ALERT_4_G Anion/Solvent/Minor-Residue Disorder (Resd  7  )       100% Note
PLAT302_ALERT_4_G Anion/Solvent/Minor-Residue Disorder (Resd  9  )       100% Note
PLAT302_ALERT_4_G Anion/Solvent/Minor-Residue Disorder (Resd 10  )       100% Note
PLAT302_ALERT_4_G Anion/Solvent/Minor-Residue Disorder (Resd 11  )       100% Note
PLAT302_ALERT_4_G Anion/Solvent/Minor-Residue Disorder (Resd 12  )       100% Note
PLAT302_ALERT_4_G Anion/Solvent/Minor-Residue Disorder (Resd 13  )       100% Note
PLAT304_ALERT_4_G Non-Integer Number of Atoms in ..... (Resd  3  )       0.75 Check
PLAT304_ALERT_4_G Non-Integer Number of Atoms in ..... (Resd  4  )       0.50 Check
PLAT304_ALERT_4_G Non-Integer Number of Atoms in ..... (Resd  5  )       0.25 Check
PLAT304_ALERT_4_G Non-Integer Number of Atoms in ..... (Resd  6  )       0.50 Check
PLAT304_ALERT_4_G Non-Integer Number of Atoms in ..... (Resd  7  )       0.25 Check
PLAT304_ALERT_4_G Non-Integer Number of Atoms in ..... (Resd  9  )       0.12 Check
PLAT304_ALERT_4_G Non-Integer Number of Atoms in ..... (Resd 10  )       0.17 Check
PLAT304_ALERT_4_G Non-Integer Number of Atoms in ..... (Resd 11  )       0.25 Check
PLAT304_ALERT_4_G Non-Integer Number of Atoms in ..... (Resd 12  )       0.12 Check
PLAT304_ALERT_4_G Non-Integer Number of Atoms in ..... (Resd 13  )       0.08 Check
PLAT311_ALERT_2_G Isolated Disordered Oxygen Atom (No H's ?) .....        O2W Check
PLAT311_ALERT_2_G Isolated Disordered Oxygen Atom (No H's ?) .....        O5W Check
PLAT311_ALERT_2_G Isolated Disordered Oxygen Atom (No H's ?) .....        O6W Check
```

```
PLAT311_ALERT_2_G Isolated Disordered Oxygen Atom (No H's ?) .....        O4W Check
PLAT311_ALERT_2_G Isolated Disordered Oxygen Atom (No H's ?) .....        O8W Check
PLAT311_ALERT_2_G Isolated Disordered Oxygen Atom (No H's ?) .....        O9W Check
PLAT311_ALERT_2_G Isolated Disordered Oxygen Atom (No H's ?) .....       O11W Check
PLAT311_ALERT_2_G Isolated Disordered Oxygen Atom (No H's ?) .....       O12W Check
PLAT395_ALERT_2_G Deviating  X-O-Y   Angle From 120 for O3W      .       85.6 Degree
PLAT395_ALERT_2_G Deviating  X-O-Y   Angle From 120 for O1W      .       60.0 Degree
PLAT432_ALERT_2_G Short Inter X...Y Contact  O2       ..C25      .       2.99 Ang.
                                             z,1-x,1/2-y  =     14_565 Check
PLAT432_ALERT_2_G Short Inter X...Y Contact  O5W      ..C23      .       3.01 Ang.
                                             z,1-x,1/2-y  =     14_565 Check
PLAT764_ALERT_4_G Overcomplete CIF Bond List Detected (Rep/Expd) .       1.11 Ratio
PLAT860_ALERT_3_G Number of Least-Squares Restraints ............          24 Note
PLAT883_ALERT_1_G No Info/Value for _atom_sites_solution_primary .     Please Do !
PLAT910_ALERT_3_G Missing # of FCF Reflection(s) Below Theta(Min).          1 Note
PLAT933_ALERT_2_G Number of HKL-OMIT Records in Embedded .res File          6 Note
PLAT955_ALERT_1_G Reported (CIF) and Actual (FCF) Lmax Differ by .          1 Units
PLAT961_ALERT_5_G Dataset Contains no Negative Intensities .......     Please Check
PLAT965_ALERT_2_G The SHELXL WEIGHT Optimisation has not Converged     Please Check
```

---

  0 **ALERT level A** = Most likely a serious problem - resolve or explain
  3 **ALERT level B** = A potentially serious problem, consider carefully
 12 **ALERT level C** = Check. Ensure it is not caused by an omission or oversight
 60 **ALERT level G** = General information/check it is not something unexpected

```
  9 ALERT type 1 CIF construction/syntax error, inconsistent or missing data
 27 ALERT type 2 Indicator that the structure model may be wrong or deficient
  3 ALERT type 3 Indicator that the structure quality may be low
 35 ALERT type 4 Improvement, methodology, query or suggestion
  1 ALERT type 5 Informative message, check
```

It is advisable to attempt to resolve as many as possible of the alerts in all categories. Often the minor alerts point to easily fixed oversights, errors and omissions in your CIF or refinement strategy, so attention to these fine details can be worthwhile. In order to resolve some of the more serious problems it may be necessary to carry out additional measurements or structure refinements. However, the purpose of your study may justify the reported deviations and the more serious of these should normally be commented upon in the discussion or experimental section of a paper or in the "special_details" fields of the CIF. checkCIF was carefully designed to identify outliers and unusual parameters, but every test has its limitations and alerts that are not important in a particular case may appear. Conversely, the absence of alerts does not guarantee there are no aspects of the results needing attention. It is up to the individual to critically assess their own results and, if necessary, seek expert advice.

Publication of your CIF in IUCr journals

A basic structural check has been run on your CIF. These basic checks will be run on all CIFs submitted for publication in IUCr journals (*Acta Crystallographica*, *Journal of Applied Crystallography*, *Journal of Synchrotron Radiation*); however, if you intend to submit to *Acta Crystallographica Section C* or *E* or *IUCrData*, you should make sure that full publication checks are run on the final version of your CIF prior to submission.

Publication of your CIF in other journals

Please refer to the *Notes for Authors* of the relevant journal for any special instructions relating to CIF submission.

PLATON version of 06/07/2023; check.def file version of 30/06/2023

Datablock jul167-4 - ellipsoid plot

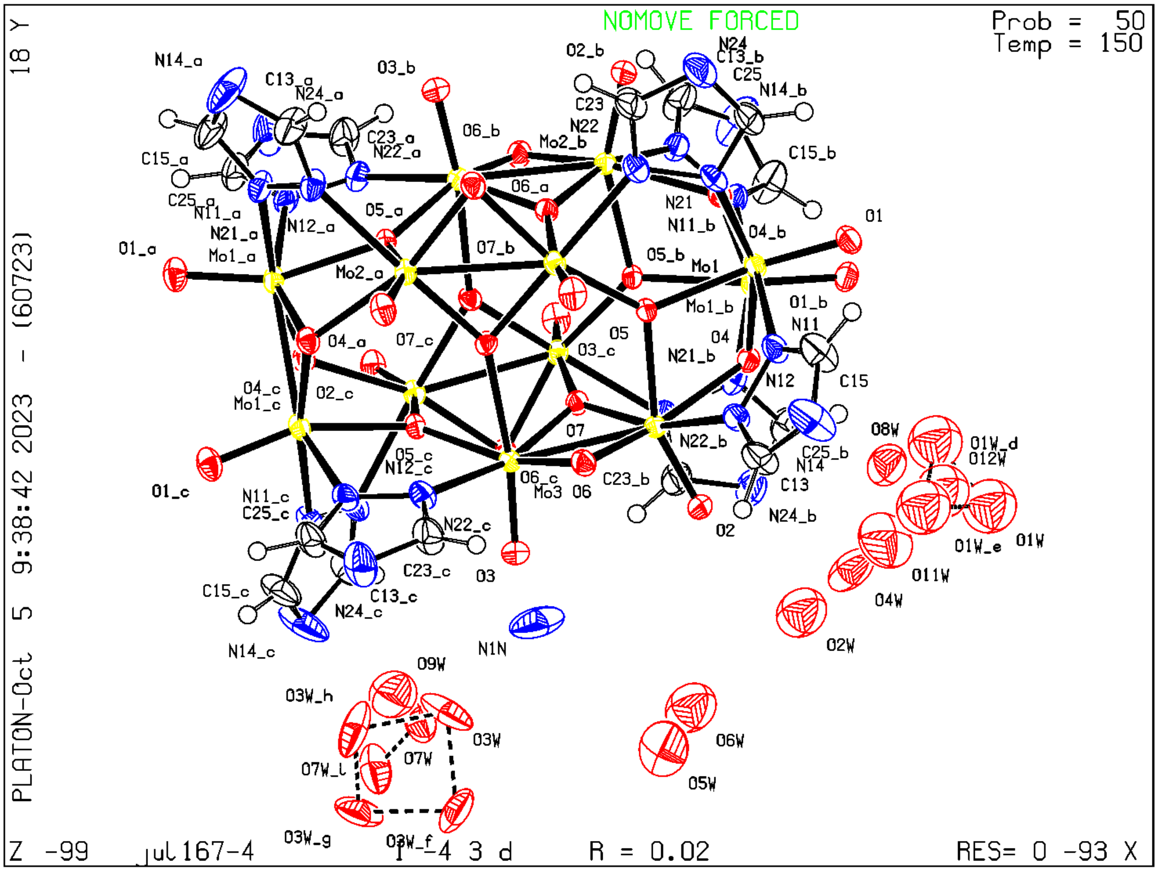

Supplement: Supplementary file 1 [file molecules-28-08079-s001.zip › Compound 2 checkcif.pdf]
