# Supplementary material for: Water-Soluble Polyoxometal Clusters of Molybdenum (V) with Pyrazole and Triazole: Synthesis and Study of Cytotoxicity and Antiviral Activity
Source: Molecules. 2023 Dec 14;28(24):8079. doi: 10.3390/molecules28248079 (PMC10745505; doi:10.3390/molecules28248079)

checkCIF/PLATON report

Structure factors have been supplied for datablock(s) jul167-3

THIS REPORT IS FOR GUIDANCE ONLY. IF USED AS PART OF A REVIEW PROCEDURE FOR PUBLICATION, IT SHOULD NOT REPLACE THE EXPERTISE OF AN EXPERIENCED CRYSTALLOGRAPHIC REFEREE.

No syntax errors found. CIF dictionary Interpreting this report

Datablock: jul167-3

Bond precision:	C-C = 0.0064 A		Wavelength=0.71073
Cell:	a=11.7496(4)	b=21.5118(7)	c=22.6938(7)
	alpha=90	beta=102.174(1)	gamma=90
Temperature:	150 K		

	Calculated	Reported
Volume	5607.0(3)	5607.0(3)
Space group	P 21/n	P 21/n
Hall group	-P 2yn	-P 2yn
Moiety formula	C16 H16 Mo12 N24 O28, 4(O), 4(N)	C16 H16 Mo12 N24 O28, 4(H2 O), 4(N H4)
Sum formula	C16 H16 Mo12 N28 O32	C16 H40 Mo12 N28 O32
Mr	2263.85	2288.04
Dx,g cm-3	2.682	2.710
Z	4	4
Mu (mm-1)	2.695	2.696
F000	4272.0	4368.0
F000'	4190.75	
h,k,lmax	18,33,34	18,33,34
Nref	21393	21345
Tmin,Tmax	0.772,0.806	0.612,0.747
Tmin'	0.764	

Correction method= # Reported T Limits: Tmin=0.612 Tmax=0.747
AbsCorr = MULTI-SCAN

Data completeness= 0.998 Theta(max)= 33.158

R(reflections)= 0.0385(16050) wR2(reflections)= 0.0914(21345)

S = 1.063 Npar= 832

The following ALERTS were generated. Each ALERT has the format
test-name_ALERT_alert-type_alert-level.
Click on the hyperlinks for more details of the test.

Alert level B

```
PLAT043_ALERT_1_B Calculated and Reported Mol. Weight Differ by ..      24.19 Check
```

Author Response: Reported formula contain 24 unlocalized H atoms of 4 disordered solvate water molecules and 4 ammonia cations.

```
PLAT430_ALERT_2_B Short Inter D...A Contact  O1WA      ..N2N      .       2.75 Ang.
                                                     x,y,z  =      1_555 Check
```

Author Response: The H atoms of the disordered solvate water molecules and ammonia cations are not localized. This contact corresponds to the H bond.

```
PLAT430_ALERT_2_B Short Inter D...A Contact  O1WA      ..N3N      .       2.77 Ang.
                                                     x,y,z  =      1_555 Check
```

Author Response: The H atoms of the disordered solvate water molecules and ammonia cations are not localized. This contact corresponds to the H bond.

```
PLAT430_ALERT_2_B Short Inter D...A Contact  O1WA      ..O25      .       2.82 Ang.
                                                     x,y,z  =      1_555 Check
```

Author Response: The H atoms of the disordered solvate water molecules and ammonia cations are not localized. This contact corresponds to the H bond.

```
PLAT430_ALERT_2_B Short Inter D...A Contact  O4        ..O2WB     .       2.82 Ang.
                                    1/2-x,-1/2+y,3/2-z  =      2_546 Check
```

Author Response: The H atoms of the disordered solvate water molecules and ammonia cations are not localized. This contact corresponds to the H bond.

```
PLAT430_ALERT_2_B Short Inter D...A Contact  O2WB      ..O9       .       2.76 Ang.
                                                  -1+x,y,z  =      1_455 Check
```

Author Response: The H atoms of the disordered solvate water molecules and ammonia cations are not localized. This contact corresponds to the H bond.

```
PLAT430_ALERT_2_B Short Inter D...A Contact  O5        ..O4WB     .       2.79 Ang.
                                                     x,y,z  =      1_555 Check
```

Author Response: The H atoms of the disordered solvate water molecules and ammonia cations are not localized. This contact corresponds to the H bond.

```
PLAT430_ALERT_2_B Short Inter D...A Contact  O3WA      ..N73       .       2.70 Ang.
                                   3/2-x,1/2+y,3/2-z  =      2_656 Check
```

Author Response: The H atoms of the disordered solvate water molecules and ammonia cations are not localized. This contact corresponds to the H bond.

```
PLAT430_ALERT_2_B Short Inter D...A Contact  O7        ..N4NB      .       2.80 Ang.
                                                x,y,z  =      1_555 Check
```

Author Response: The H atoms of the disordered solvate water molecules and ammonia cations are not localized. This contact corresponds to the H bond.

```
PLAT430_ALERT_2_B Short Inter D...A Contact  O7        ..N1N       .       2.86 Ang.
                                                x,y,z  =      1_555 Check
```

Author Response: The H atoms of the disordered solvate water molecules and ammonia cations are not localized. This contact corresponds to the H bond.

```
PLAT430_ALERT_2_B Short Inter D...A Contact  O9        ..N4NA      .       2.79 Ang.
                                              1+x,y,z  =      1_655 Check
```

Author Response: The H atoms of the disordered solvate water molecules and ammonia cations are not localized. This contact corresponds to the H bond.

```
PLAT430_ALERT_2_B Short Inter D...A Contact  O25       ..O1WB      .       2.72 Ang.
                                                x,y,z  =      1_555 Check
```

Author Response: The H atoms of the disordered solvate water molecules and ammonia cations are not localized. This contact corresponds to the H bond.

```
PLAT430_ALERT_2_B Short Inter D...A Contact  O26       ..N4NA      .       2.59 Ang.
                                                x,y,z  =      1_555 Check
```

Author Response: The H atoms of the disordered solvate water molecules and ammonia cations are not localized. This contact corresponds to the H bond.

```
PLAT430_ALERT_2_B Short Inter D...A Contact  O27       ..N1N       .       2.76 Ang.
                                  1/2-x,-1/2+y,3/2-z  =      2_546 Check
```

Author Response: The H atoms of the disordered solvate water molecules and ammonia cations are not localized. This contact corresponds to the H bond.

```
PLAT430_ALERT_2_B Short Inter D...A Contact  O28       ..N3N      .       2.71 Ang.
                                 3/2-x,-1/2+y,3/2-z  =      2_646 Check
```

Author Response: The H atoms of the disordered solvate water molecules and ammonia cations are not localized. This contact corresponds to the H bond.

```
PLAT430_ALERT_2_B Short Inter D...A Contact  N1N       ..N2N      .       2.66 Ang.
                                             x,y,z  =      1_555 Check
```

Author Response: The H atoms of the disordered solvate water molecules and ammonia cations are not localized. This contact corresponds to the H bond.

```
PLAT430_ALERT_2_B Short Inter D...A Contact  O4WC      ..O4WD     .       2.80 Ang.
                                             x,y,z  =      1_555 Check
```

Author Response: The H atoms of the disordered solvate water molecules and ammonia cations are not localized. This contact corresponds to the H bond.

Alert level C

```
PLAT041_ALERT_1_C Calc. and Reported SumFormula    Strings  Differ     Please Check
PLAT068_ALERT_1_C Reported F000 Differs from Calcd (or Missing)...     Please Check
PLAT230_ALERT_2_C Hirshfeld Test Diff for    C34      --C35      .        6.8 s.u.
PLAT241_ALERT_2_C High   'MainMol' Ueq as Compared to Neighbors of        C54 Check
PLAT411_ALERT_2_C Short Inter H...H Contact  H25A      ..H65      .       2.12 Ang.
                                -1/2+x,1/2-y,-1/2+z  =      4_565 Check
PLAT430_ALERT_2_C Short Inter D...A Contact  O5        ..O4WC     .       2.87 Ang.
                                             x,y,z  =      1_555 Check
```

Author Response: The H atoms of the disordered solvate water molecules and ammonia cations are not localized. This contact corresponds to the H bond.

```
PLAT430_ALERT_2_C Short Inter D...A Contact  O8        ..O4WB     .       2.89 Ang.
                                 3/2-x,-1/2+y,3/2-z  =      2_646 Check
```

Author Response: The H atoms of the disordered solvate water molecules and ammonia cations are not localized. This contact corresponds to the H bond.

```
PLAT430_ALERT_2_C Short Inter D...A Contact  O12       ..N2N      .       2.90 Ang.
                                  1/2+x,1/2-y,1/2+z  =      4_666 Check
```

Author Response: The H atoms of the disordered solvate water molecules and ammonia cations are not localized. This contact corresponds to the H bond.

```
PLAT906_ALERT_3_C Large K Value in the Analysis of Variance ......      3.148 Check
PLAT911_ALERT_3_C Missing FCF Refl Between Thmin & STh/L=    0.600         18 Report
PLAT972_ALERT_2_C Check Calcd Resid. Dens.  0.62Ang From Mo11           -1.72 eA-3
PLAT973_ALERT_2_C Check Calcd Positive Resid. Density on      Mo11       1.02 eA-3
PLAT975_ALERT_2_C Check Calcd Resid. Dens.  0.77Ang From N1N     .       1.04 eA-3
PLAT975_ALERT_2_C Check Calcd Resid. Dens.  0.76Ang From N2N     .       1.03 eA-3
PLAT975_ALERT_2_C Check Calcd Resid. Dens.  0.87Ang From O4WD    .       0.84 eA-3
PLAT975_ALERT_2_C Check Calcd Resid. Dens.  0.76Ang From N3N     .       0.82 eA-3
PLAT977_ALERT_2_C Check Negative Difference Density on H14       .      -0.45 eA-3
PLAT977_ALERT_2_C Check Negative Difference Density on H25A      .      -0.38 eA-3
PLAT977_ALERT_2_C Check Negative Difference Density on H35       .      -0.43 eA-3
PLAT977_ALERT_2_C Check Negative Difference Density on H65       .      -0.57 eA-3
PLAT977_ALERT_2_C Check Negative Difference Density on H83       .      -0.43 eA-3
```

Alert level G

```
FORMU01_ALERT_2_G  There is a discrepancy between the atom counts in the
            _chemical_formula_sum and the formula from the _atom_site* data.
            Atom count from _chemical_formula_sum:C16 H40 Mo12 N28 O32
            Atom count from the _atom_site data:  C16 H16 Mo12 N28 O32
CELLZ01_ALERT_1_G Difference between formula and atom_site contents detected.
CELLZ01_ALERT_1_G WARNING: H atoms missing from atom site list. Is this intentional?
           From the CIF: _cell_formula_units_Z    4
           From the CIF: _chemical_formula_sum  C16 H40 Mo12 N28 O32
           TEST: Compare cell contents of formula and atom_site data

           atom    Z*formula  cif sites diff
           C         64.00     64.00    0.00
           H        160.00     64.00   96.00
           Mo        48.00     48.00    0.00
           N        112.00    112.00    0.00
           O        128.00    128.00    0.00
PLAT003_ALERT_2_G Number of Uiso or Uij Restrained non-H Atoms ...          5 Report
PLAT171_ALERT_4_G The CIF-Embedded .res File Contains EADP Records          4 Report
PLAT186_ALERT_4_G The CIF-Embedded .res File Contains ISOR Records          2 Report
PLAT300_ALERT_4_G Atom Site Occupancy of O1WA       Constrained at       0.75 Check
PLAT300_ALERT_4_G Atom Site Occupancy of O2WA       Constrained at        0.5 Check
PLAT300_ALERT_4_G Atom Site Occupancy of O2WB       Constrained at        0.5 Check
PLAT300_ALERT_4_G Atom Site Occupancy of O3WA       Constrained at        0.5 Check
PLAT300_ALERT_4_G Atom Site Occupancy of O3WB       Constrained at        0.5 Check
PLAT300_ALERT_4_G Atom Site Occupancy of N4NA       Constrained at        0.5 Check
PLAT300_ALERT_4_G Atom Site Occupancy of N4NB       Constrained at        0.5 Check
PLAT300_ALERT_4_G Atom Site Occupancy of O1WB       Constrained at       0.25 Check
PLAT300_ALERT_4_G Atom Site Occupancy of O4WA       Constrained at       0.25 Check
PLAT300_ALERT_4_G Atom Site Occupancy of O4WB       Constrained at       0.25 Check
PLAT300_ALERT_4_G Atom Site Occupancy of O4WC       Constrained at       0.25 Check
PLAT300_ALERT_4_G Atom Site Occupancy of O4WD       Constrained at       0.25 Check
PLAT302_ALERT_4_G Anion/Solvent/Minor-Residue Disorder (Resd  2  )       100% Note
PLAT302_ALERT_4_G Anion/Solvent/Minor-Residue Disorder (Resd  3  )       100% Note
PLAT302_ALERT_4_G Anion/Solvent/Minor-Residue Disorder (Resd  4  )       100% Note
PLAT302_ALERT_4_G Anion/Solvent/Minor-Residue Disorder (Resd  5  )       100% Note
PLAT302_ALERT_4_G Anion/Solvent/Minor-Residue Disorder (Resd  6  )       100% Note
PLAT302_ALERT_4_G Anion/Solvent/Minor-Residue Disorder (Resd  7  )       100% Note
PLAT302_ALERT_4_G Anion/Solvent/Minor-Residue Disorder (Resd  9  )       100% Note
PLAT302_ALERT_4_G Anion/Solvent/Minor-Residue Disorder (Resd 12  )       100% Note
PLAT302_ALERT_4_G Anion/Solvent/Minor-Residue Disorder (Resd 13  )       100% Note
PLAT302_ALERT_4_G Anion/Solvent/Minor-Residue Disorder (Resd 14  )       100% Note
```

```
PLAT302_ALERT_4_G Anion/Solvent/Minor-Residue Disorder (Resd 15  )       100% Note
PLAT302_ALERT_4_G Anion/Solvent/Minor-Residue Disorder (Resd 16  )       100% Note
PLAT304_ALERT_4_G Non-Integer Number of Atoms in ..... (Resd  2  )       0.75 Check
PLAT304_ALERT_4_G Non-Integer Number of Atoms in ..... (Resd  3  )       0.50 Check
PLAT304_ALERT_4_G Non-Integer Number of Atoms in ..... (Resd  4  )       0.50 Check
PLAT304_ALERT_4_G Non-Integer Number of Atoms in ..... (Resd  5  )       0.50 Check
PLAT304_ALERT_4_G Non-Integer Number of Atoms in ..... (Resd  6  )       0.50 Check
PLAT304_ALERT_4_G Non-Integer Number of Atoms in ..... (Resd  7  )       0.50 Check
PLAT304_ALERT_4_G Non-Integer Number of Atoms in ..... (Resd  9  )       0.50 Check
PLAT304_ALERT_4_G Non-Integer Number of Atoms in ..... (Resd 12  )       0.25 Check
PLAT304_ALERT_4_G Non-Integer Number of Atoms in ..... (Resd 13  )       0.25 Check
PLAT304_ALERT_4_G Non-Integer Number of Atoms in ..... (Resd 14  )       0.25 Check
PLAT304_ALERT_4_G Non-Integer Number of Atoms in ..... (Resd 15  )       0.25 Check
PLAT304_ALERT_4_G Non-Integer Number of Atoms in ..... (Resd 16  )       0.25 Check
PLAT311_ALERT_2_G Isolated Disordered Oxygen Atom (No H's ?) .....       O1WA Check
PLAT311_ALERT_2_G Isolated Disordered Oxygen Atom (No H's ?) .....       O2WA Check
PLAT311_ALERT_2_G Isolated Disordered Oxygen Atom (No H's ?) .....       O2WB Check
PLAT311_ALERT_2_G Isolated Disordered Oxygen Atom (No H's ?) .....       O3WA Check
PLAT311_ALERT_2_G Isolated Disordered Oxygen Atom (No H's ?) .....       O3WB Check
PLAT311_ALERT_2_G Isolated Disordered Oxygen Atom (No H's ?) .....       O1WB Check
PLAT311_ALERT_2_G Isolated Disordered Oxygen Atom (No H's ?) .....       O4WA Check
PLAT311_ALERT_2_G Isolated Disordered Oxygen Atom (No H's ?) .....       O4WB Check
PLAT311_ALERT_2_G Isolated Disordered Oxygen Atom (No H's ?) .....       O4WC Check
PLAT311_ALERT_2_G Isolated Disordered Oxygen Atom (No H's ?) .....       O4WD Check
PLAT432_ALERT_2_G Short Inter X...Y Contact  O2       ..C55      .       3.00 Ang.
                                        -1/2+x,1/2-y,-1/2+z  =      4_565 Check
PLAT720_ALERT_4_G Number of Unusual/Non-Standard Labels ..........         12 Note
PLAT860_ALERT_3_G Number of Least-Squares Restraints .............         30 Note
PLAT883_ALERT_1_G No Info/Value for _atom_sites_solution_primary .     Please Do !
PLAT898_ALERT_4_G Second Reported H-M Symbol in CIF Ignored ......          ! Check
PLAT910_ALERT_3_G Missing # of FCF Reflection(s) Below Theta(Min).          3 Note
PLAT912_ALERT_4_G Missing # of FCF Reflections Above STh/L=  0.600         28 Note
PLAT913_ALERT_3_G Missing # of Very Strong Reflections in FCF ....          1 Note
PLAT933_ALERT_2_G Number of HKL-OMIT Records in Embedded .res File         15 Note
PLAT941_ALERT_3_G Average HKL Measurement Multiplicity ...........        3.5 Low
PLAT961_ALERT_5_G Dataset Contains no Negative Intensities .......     Please Check
PLAT978_ALERT_2_G Number C-C Bonds with Positive Residual Density.          0 Info
```

```
  0 ALERT level A = Most likely a serious problem - resolve or explain
 17 ALERT level B = A potentially serious problem, consider carefully
 21 ALERT level C = Check. Ensure it is not caused by an omission or oversight
 64 ALERT level G = General information/check it is not something unexpected

  6 ALERT type 1 CIF construction/syntax error, inconsistent or missing data
 48 ALERT type 2 Indicator that the structure model may be wrong or deficient
  6 ALERT type 3 Indicator that the structure quality may be low
 41 ALERT type 4 Improvement, methodology, query or suggestion
  1 ALERT type 5 Informative message, check
```

It is advisable to attempt to resolve as many as possible of the alerts in all categories. Often the minor alerts point to easily fixed oversights, errors and omissions in your CIF or refinement strategy, so attention to these fine details can be worthwhile. In order to resolve some of the more serious problems it may be necessary to carry out additional measurements or structure refinements. However, the purpose of your study may justify the reported deviations and the more serious of these should normally be commented upon in the discussion or experimental section of a paper or in the "special_details" fields of the CIF. checkCIF was carefully designed to identify outliers and unusual parameters, but every test has its limitations and alerts that are not important in a particular case may appear. Conversely, the absence of alerts does not guarantee there are no aspects of the results needing attention. It is up to the individual to critically assess their own results and, if necessary, seek expert advice.

Publication of your CIF in IUCr journals

A basic structural check has been run on your CIF. These basic checks will be run on all CIFs submitted for publication in IUCr journals (*Acta Crystallographica*, *Journal of Applied Crystallography*, *Journal of Synchrotron Radiation*); however, if you intend to submit to *Acta Crystallographica Section C* or *E* or *IUCrData*, you should make sure that full publication checks are run on the final version of your CIF prior to submission.

Publication of your CIF in other journals

Please refer to the *Notes for Authors* of the relevant journal for any special instructions relating to CIF submission.

PLATON version of 06/07/2023; check.def file version of 30/06/2023

Datablock jul167-3 - ellipsoid plot

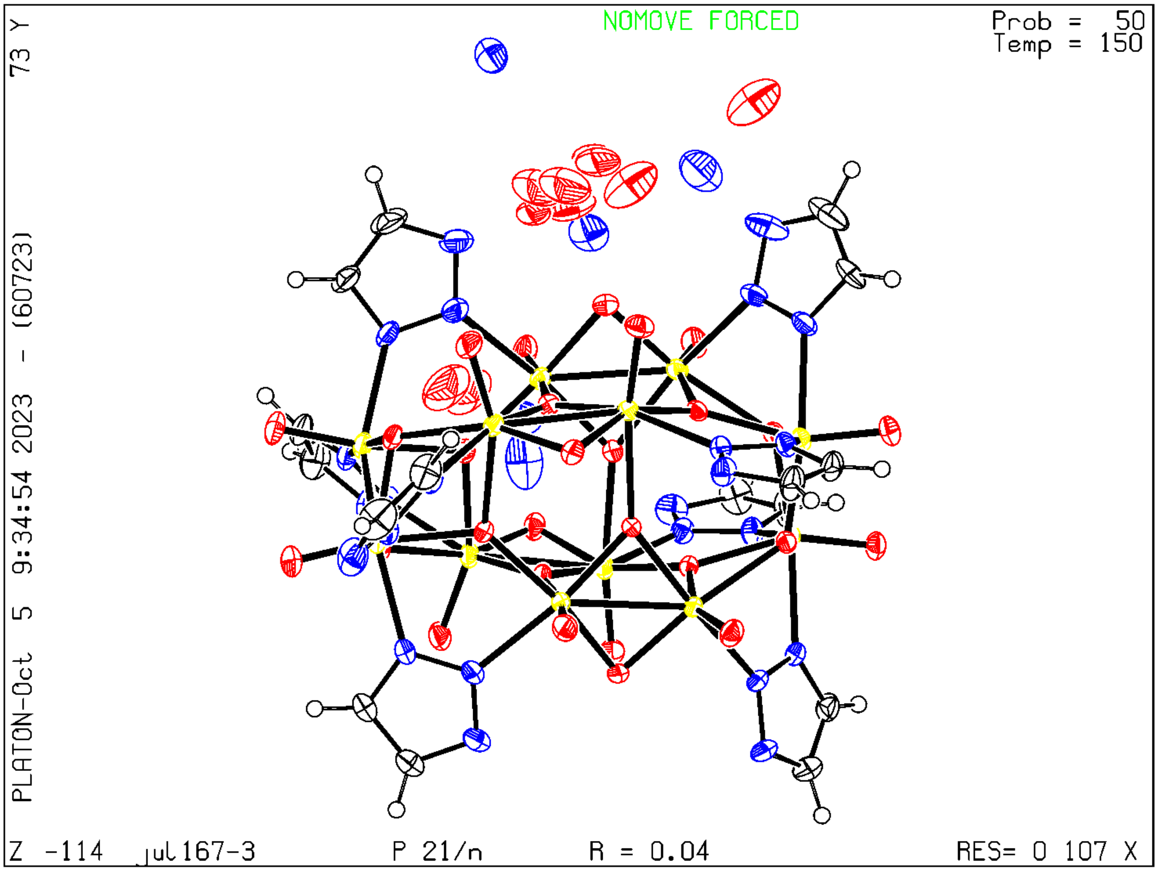

Supplement: Supplementary file 1 [file molecules-28-08079-s001.zip › Compound 3 checkcif.pdf]
