# Supplementary material for: Water-Soluble Polyoxometal Clusters of Molybdenum (V) with Pyrazole and Triazole: Synthesis and Study of Cytotoxicity and Antiviral Activity
Source: Molecules. 2023 Dec 14;28(24):8079. doi: 10.3390/molecules28248079 (PMC10745505; doi:10.3390/molecules28248079)
Supplement: Supplementary file 1 [file molecules-28-08079-s001.zip › ESI revised.pdf]

Water soluble polyoxometal clusters of molybdenum (V) with pyrazole and triazole: synthesis and study of cytotoxicity and antiviral activity

Anna V. Konkova [1], Iulia V. Savina [1], Darya V. Evtushok [1], Tatiana N. Pozmogova [1], Maria V. Solomatina [2], Alina R. Nokhova [2], Alexander Y. Alekseev [2,3], Natalia V. Kuratieva [1], Ilia V. Eltsov [4], Vadim V. Yanshole [5,6], Aleksander M. Shestopalov [2,3], Anton A. Ivanov [1,*], Michael A. Shestopalov[1]

[1] Nikolaev Institute of Inorganic Chemistry SB RAS, 3 Acad. Lavrentiev Ave., 630090 Novosibirsk, Russia; konkova@niic.nsc.ru (A.V.K.); savina@niic.nsc.ru (I.V.S.); evtushok@niic.nsc.ru (D.V.E.); pozmogova@niic.nsc.ru (T.N.P.); kuratieva@gmail.com (N.V.K.); ivanov338@niic.nsc.ru (A.A.I.); shtopy@niic.nsc.ru (M.A.S.)

[2] Research Institute of Virology, Federal Research Center of Fundamental and Translational Medicine, 2 Timakova St, 630117 Novosibirsk, Russia; mariaza@ngs.ru (M.V.S.); alina-nohova@mail.ru (A.R.N.); al-alexok@yandex.ru (A.Y.A.); shestopalov2@mail.ru (A.M.S.);

[3] Research Institute of Applied Ecology, Dagestan State University, 43a Gadzhiyeva St, 367000 Makhachkala, Dagestan, Russia; al-alexok@yandex.ru (A.Y.A.); shestopalov2@mail.ru (A.M.S.);

[4] Department of Natural Sciences, Novosibirsk State University, 2 Pirogov Str., 630090 Novosibirsk, Russia; eiv@fen.nsu.ru (I.V.E.);

[5] Department of Physics, Novosibirsk State University, 1 Pirogova st., 630090 Novosibirsk, Russia; vadim.yanshole@tomo.nsc.ru (V.V.Y.);

[6] International Tomography Center SB RAS, 3a Institutskaya Str., 630090 Novosibirsk, Russia; vadim.yanshole@tomo.nsc.ru (V.V.Y.);

* Correspondence: ivanov338@niic.nsc.ru;

TABLE OF CONTENT

Crystal structure data 3
NMR and mass-spectrometry data for 1 5
NMR and mass-spectrometry data for 2 10
NMR and mass-spectrometry data for 3 15
NMR-spectra of 2 after precipitation with HCl 16
UV-vis spectra of solutions in culture medium 17
ICP-AES data 18
IR spectra 19
Thermogravimetric analysis 21
Powder X-ray diffraction analysis 23
Crystal structure data 25
References 26

Crystal structure data

Table S1. Main interatomic distances (Å) for **1**, **2** and **3** in comparison with literature data.

Compound	Mo–Mo	Mo–μ-O	Mo–N	Ref
**1**	2.5773(11)–2.6225(12)	1.916(7)–1.990(7)	2.143(8)–2.197(9)	This study
**1**	2.5803–2.6014(9)	1.923(4)–1.981(4)	2.148(5)–2.199(5)	This study
**3**	2.5731(4)–2.6036(4)	1.913(2)–1.989(3)	2.157(3)–2.229(3)	This study
$[Mo^{V}_{6}Mo^{VI}_{2}(\mu\text{-pz})_{6}O_{18}(pzH)_{6}]$	2.5722(5)–2.5752(4)	1.928(2)–1.950(2)	μ-pz: 2.195(2)–2.206(2) pzH: 2.256(2)-2.240 (2)	1
$[Mo_{8}{}^{V}Mo_{2}{}^{VI}O_{26}(3\text{-Me-Py})_{8}]$	2.5576(8)–2.6128(8)	1.812(4)–2.050(5)	2.213(6)–2.251(6)	2

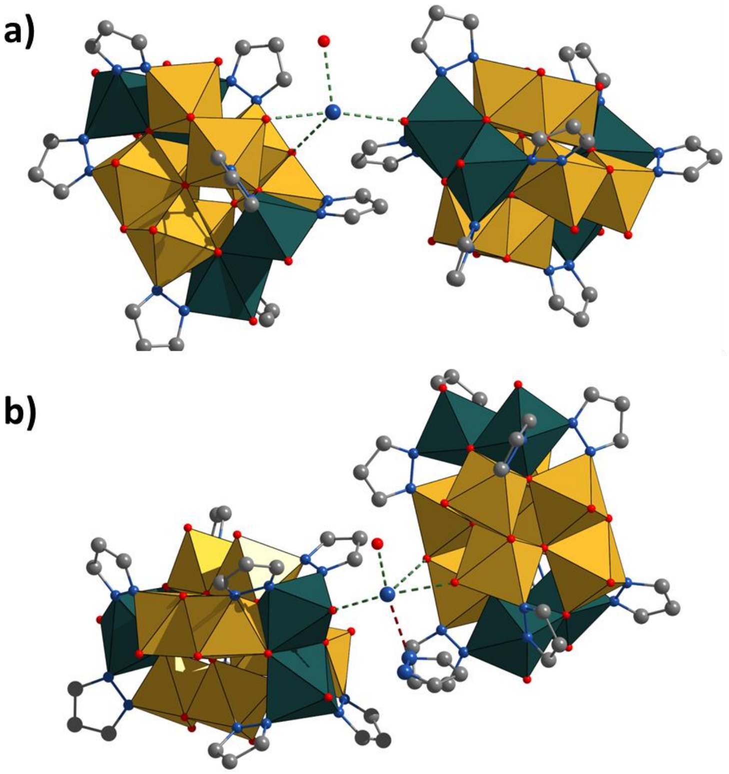

Figure S1. Hydrogen bonds observed in the crystal structure of **1**. Color code: O – red, C – gray, N – blue, $Mo^{V}{}_{2}O_{6}N_{4}$ – yellow edge-connected octahedrons, $Mo^{V}O_{8}N_{2}$ – blue-green edge-connected octahedrons, N···O – green dashed line, N···N – red dashed line.

a)

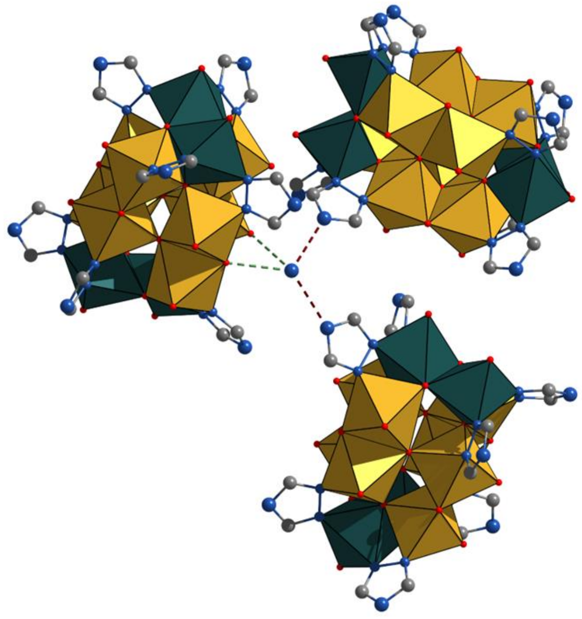

b)

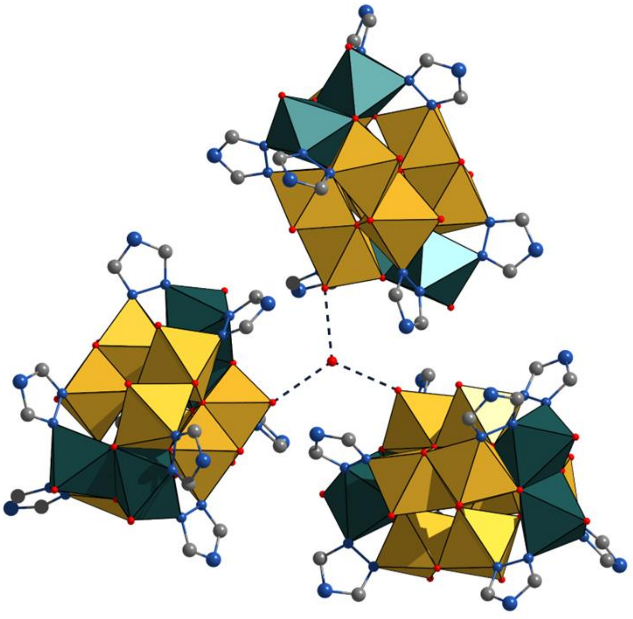

Figure S2. Hydrogen bonds observed in the crystal structure of **2**. Color code: O – red, C – gray, N – blue, $Mo^{V}{}_2O_6N_4$ – yellow edge-connected octahedrons, $Mo^{V}O_8N_2$ – blue-green edge-connected octahedrons, N···O – green dashed line, N···N – red dashed line, O···O – blue dashed line.

NMR and mass-spectrometry data for 1

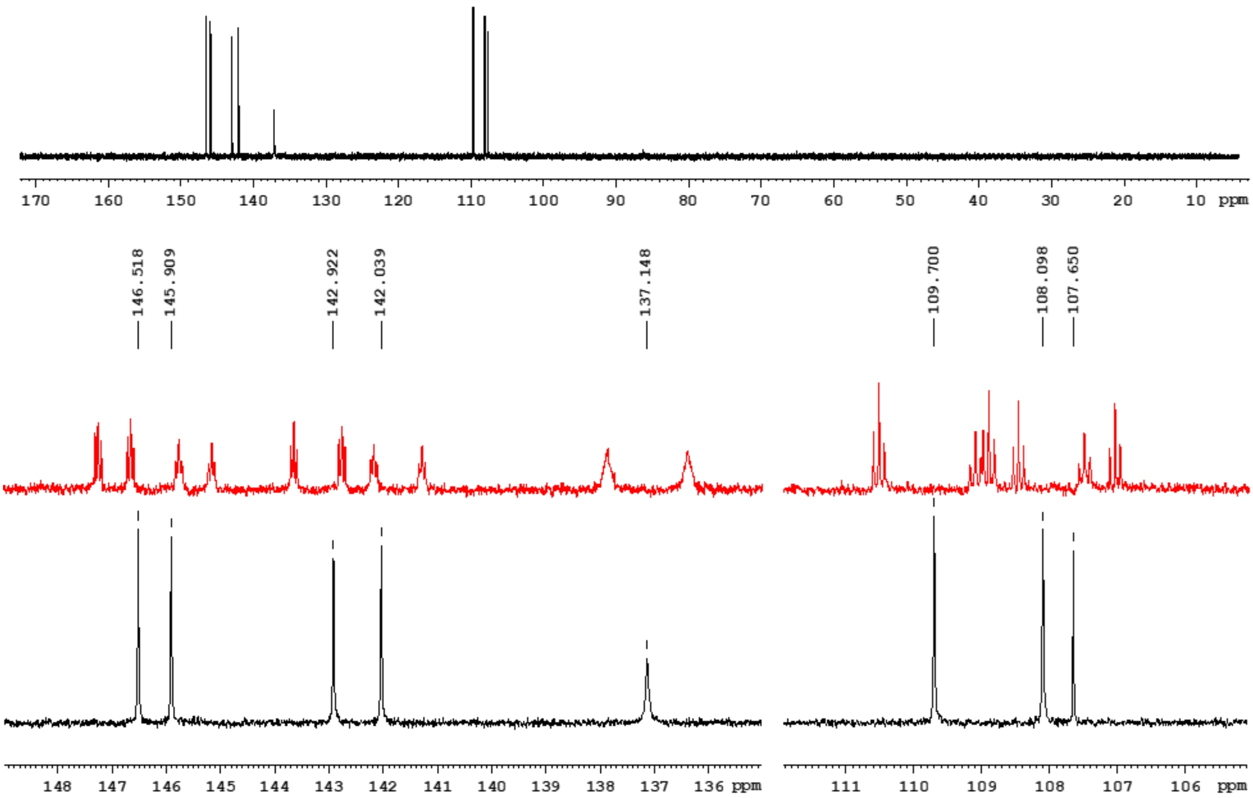

Figure S3. ^{13}C NMR (red) and ^{13}C{^{1}H} (black) spectra of **1** in D_2O.

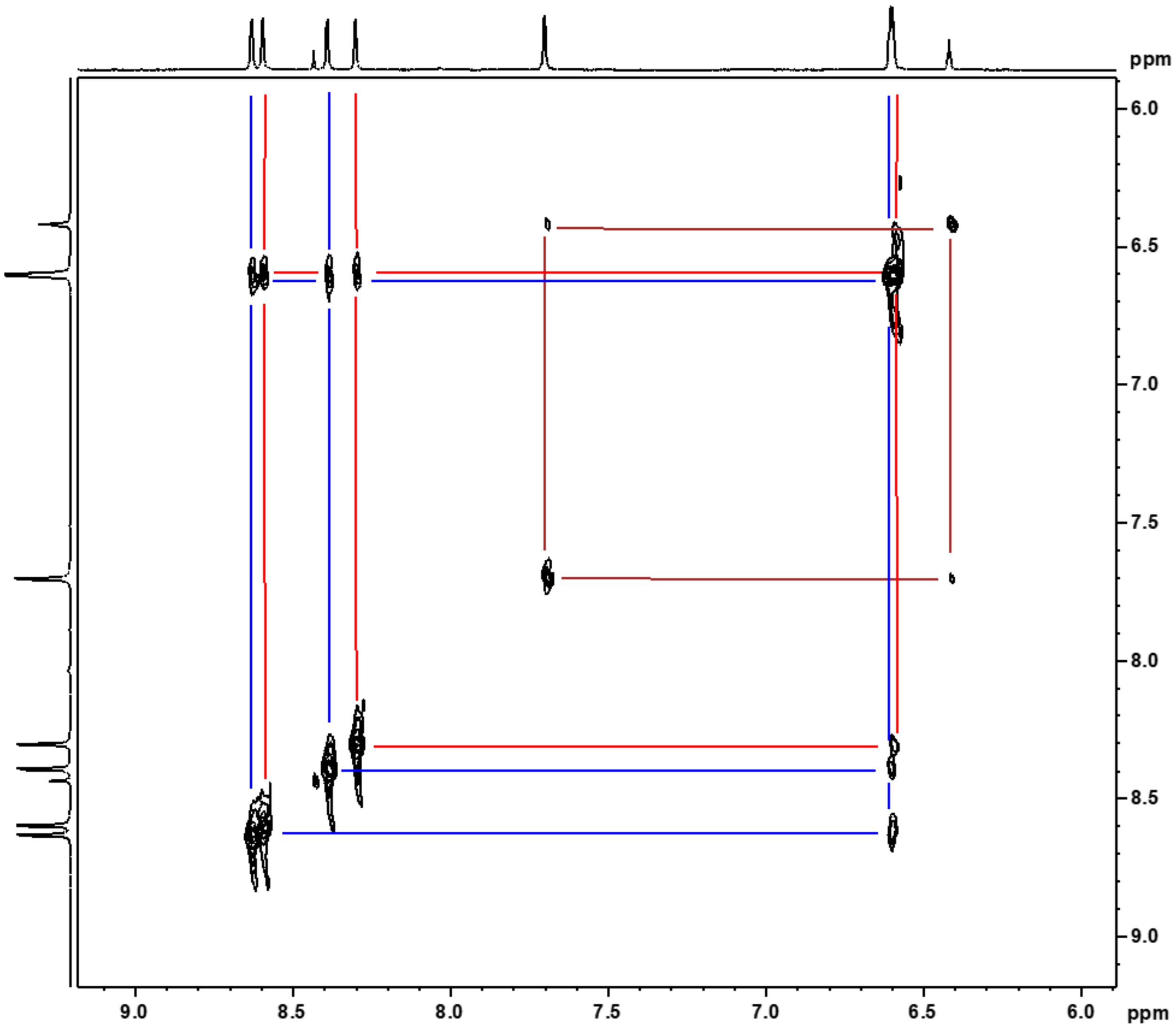

Figure S4. ^{1}H,^{1}H-NMR correlation spectra of **1** in D_2O.

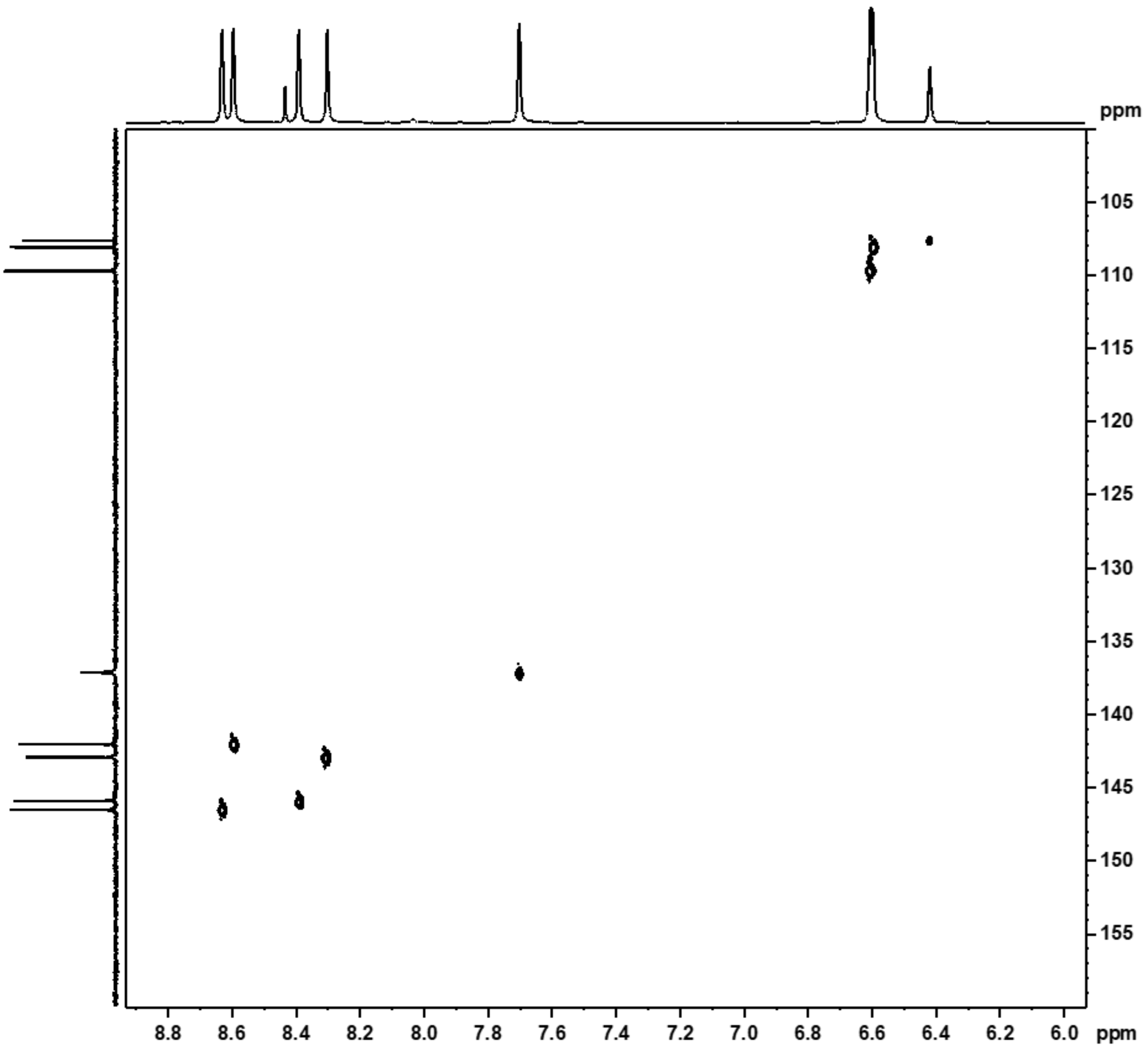

Figure S5. $^{1}H,^{13}C$-NMR correlation (HSQC) spectra of **1** in D_2O.

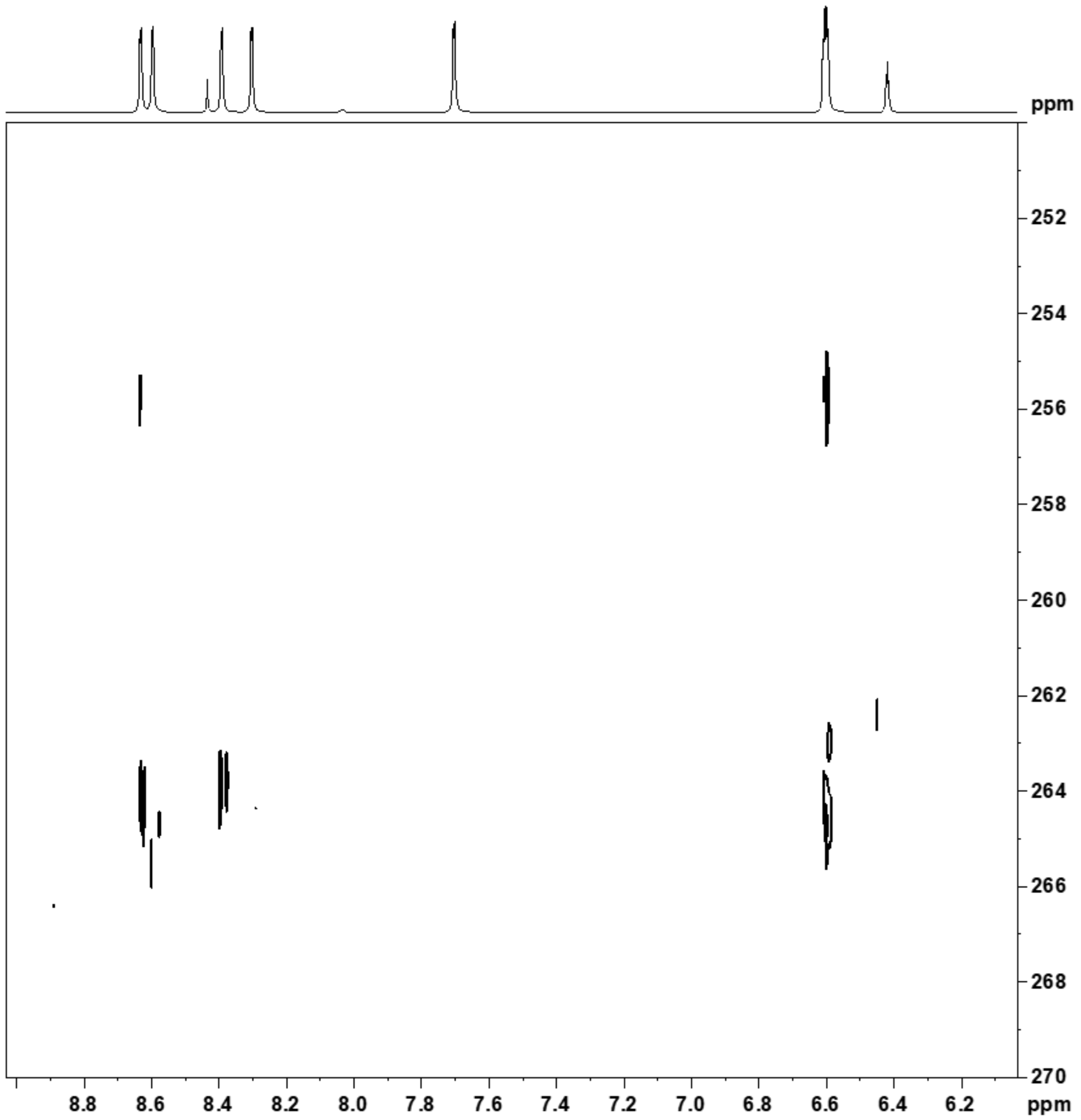

Figure S6. ^{1}H-^{15}N HMBC NMR spectra of **1** in D_2O.

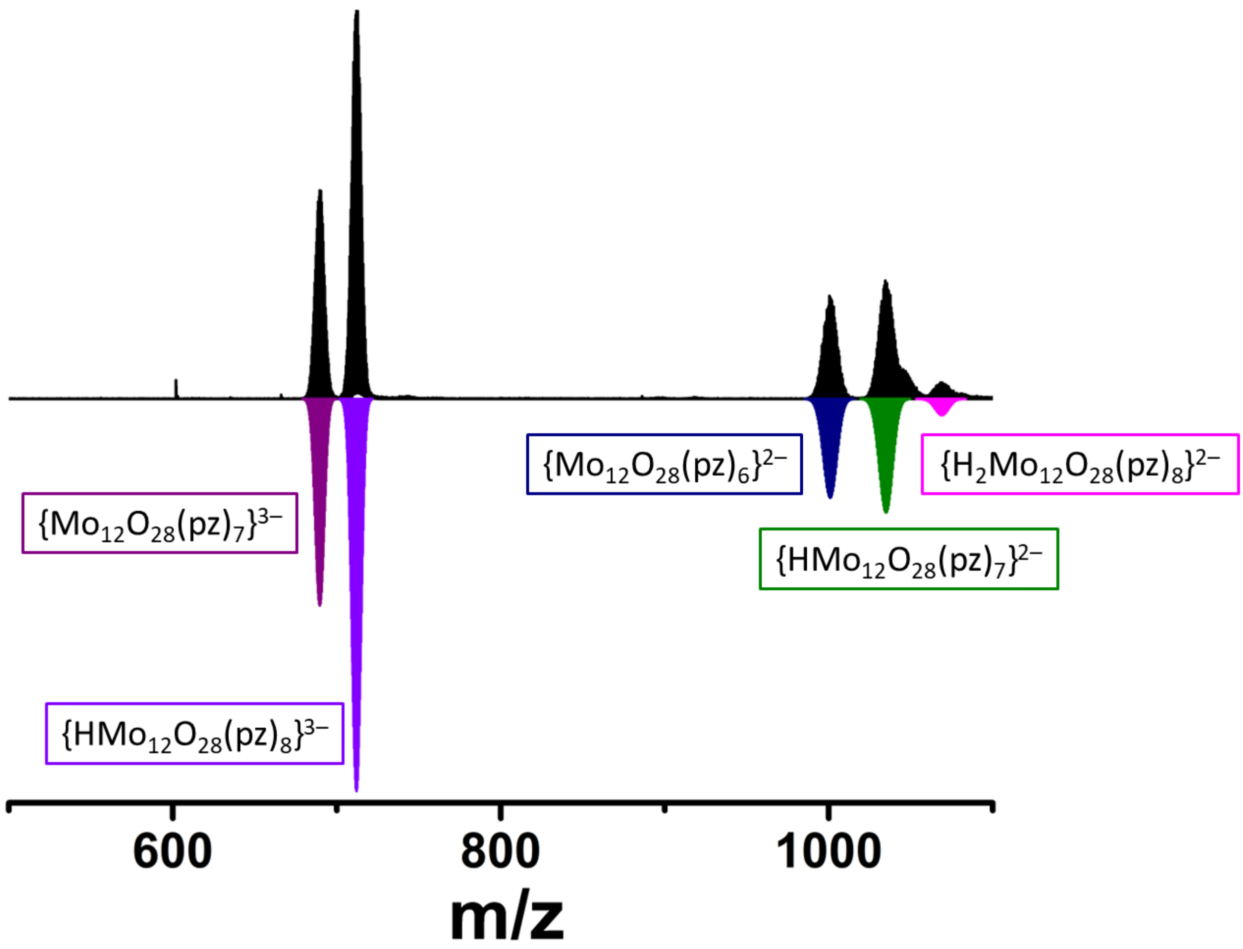

Figure S7. HR-ESI-MS spectrum of solution of **1** in water (black) and simulations of cluster forms (colored).

NMR and mass-spectrometry data for 2

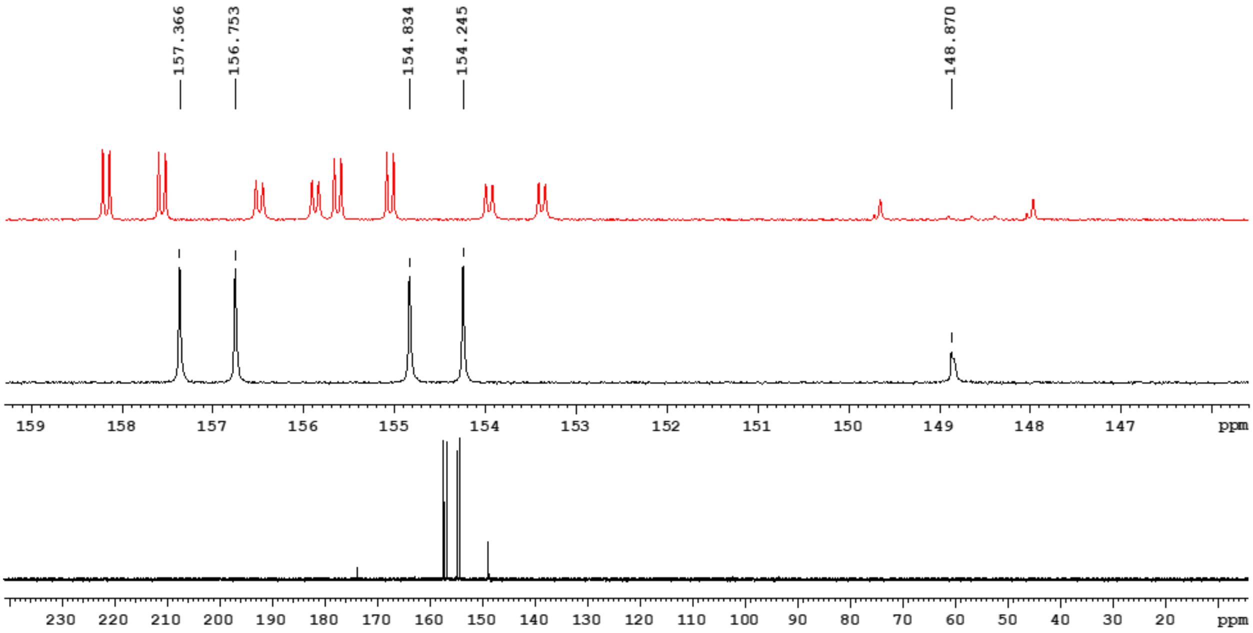

Figure S8. ^{13}C NMR (red) and $^{13}C\{^1H\}$ (black) spectra of **2** in D_2O.

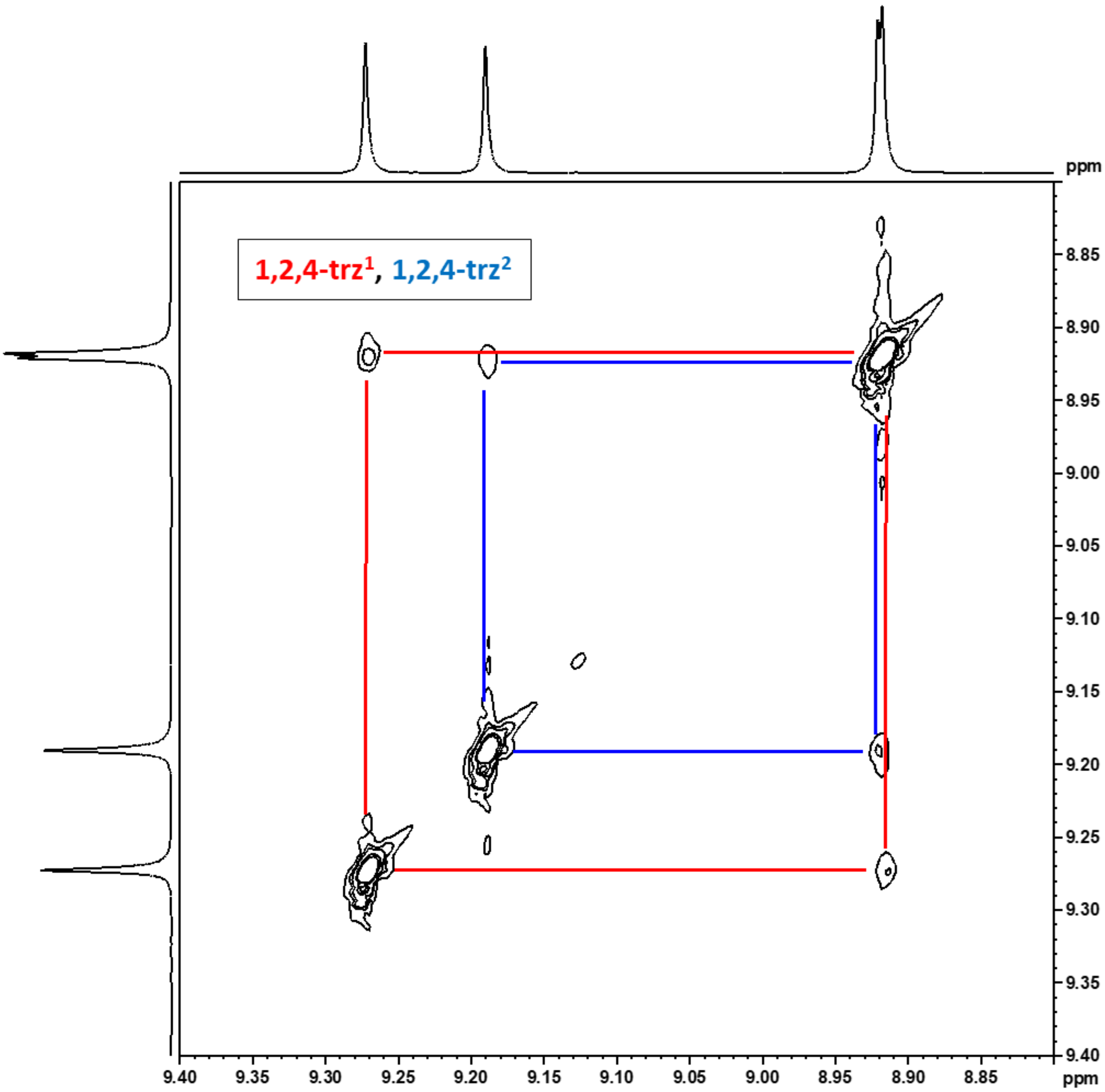

Figure S9. $^{1}H,^{1}H$-NMR correlation spectra of **2** in D_2O.

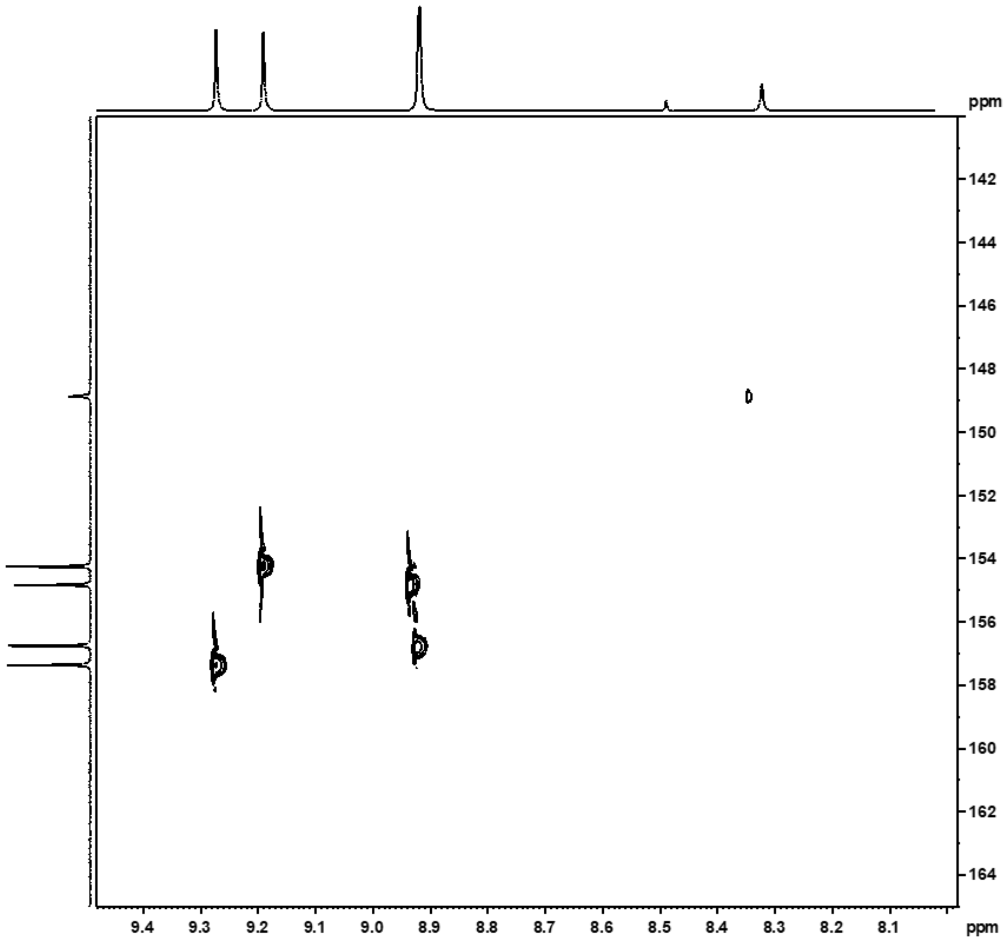

Figure S10. $^{1}H,^{13}C$-NMR correlation (HSQC) spectra of **2** in D_2O.

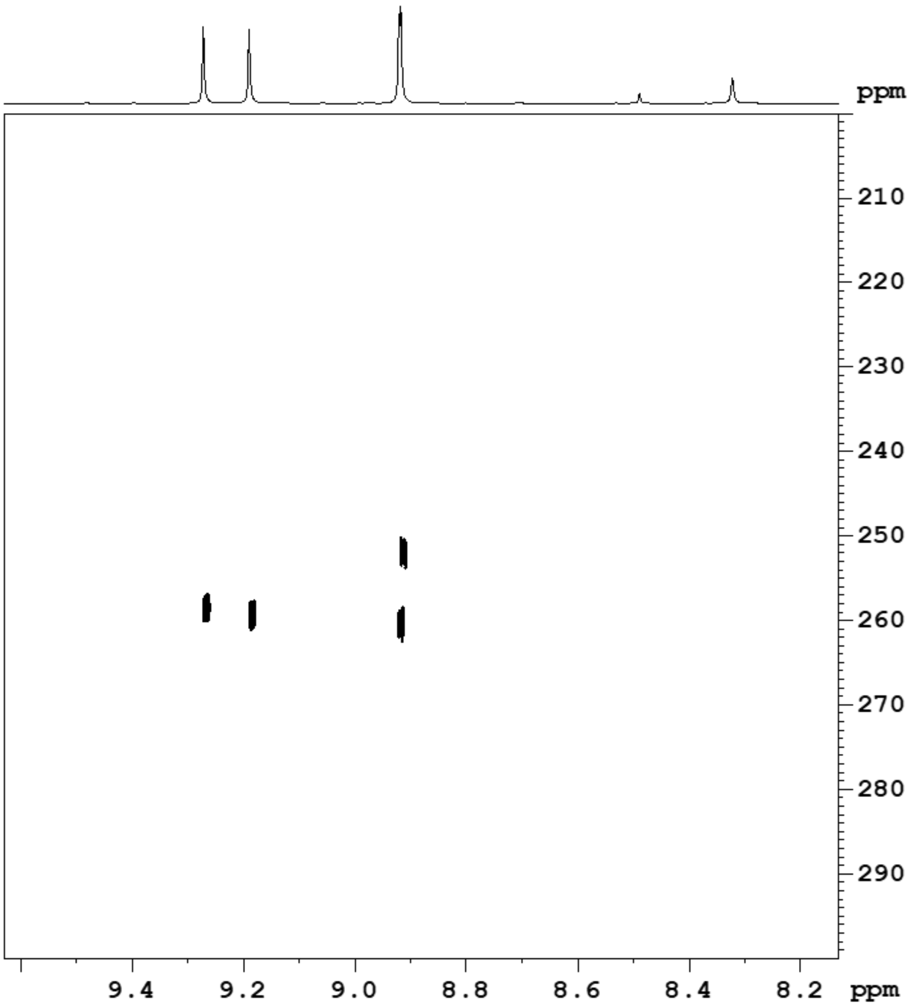

Figure S11. ^{1}H-^{15}N HMBC NMR spectra of **2** in D_2O.

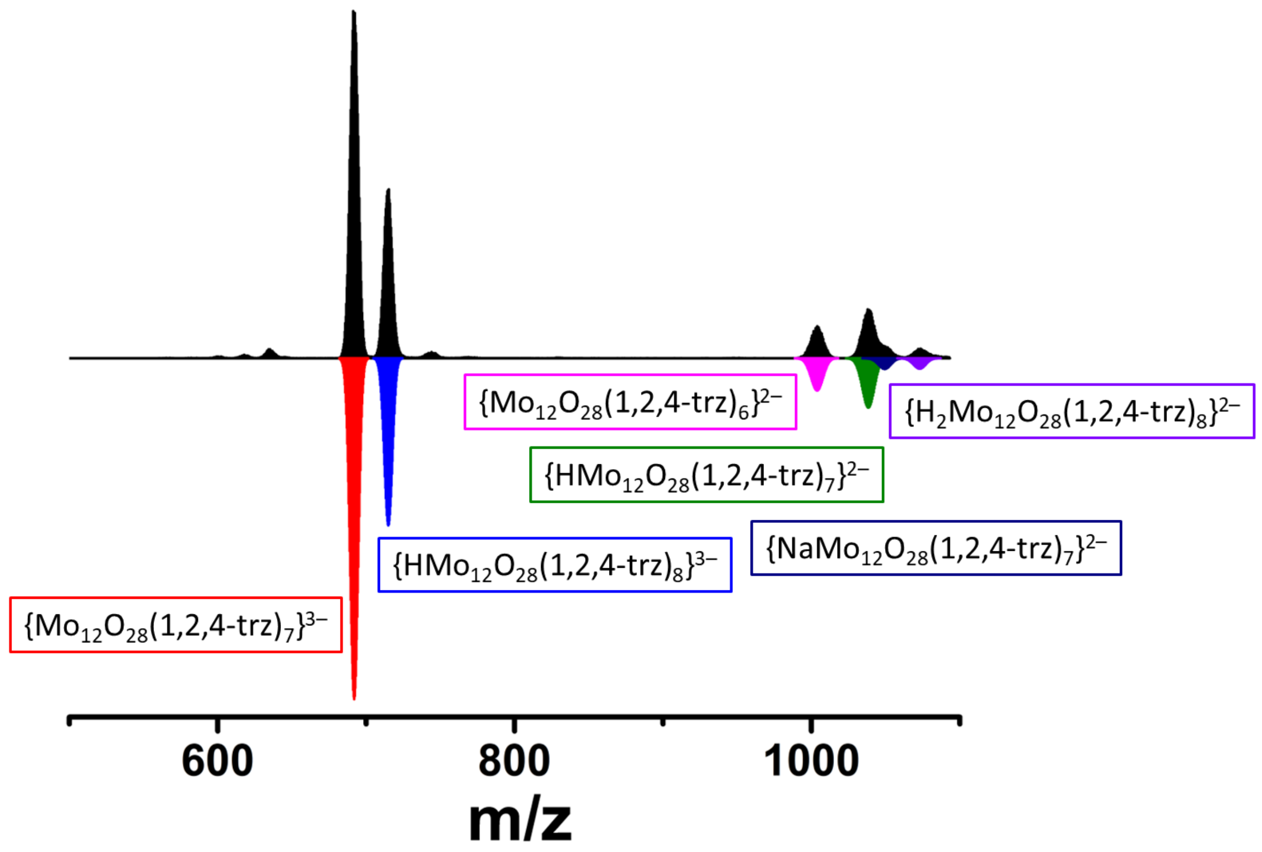

Figure S12. HR-ESI-MS spectrum of solution of **2** in water (black) and simulations of cluster forms (colored).

NMR and mass-spectrometry data for 3

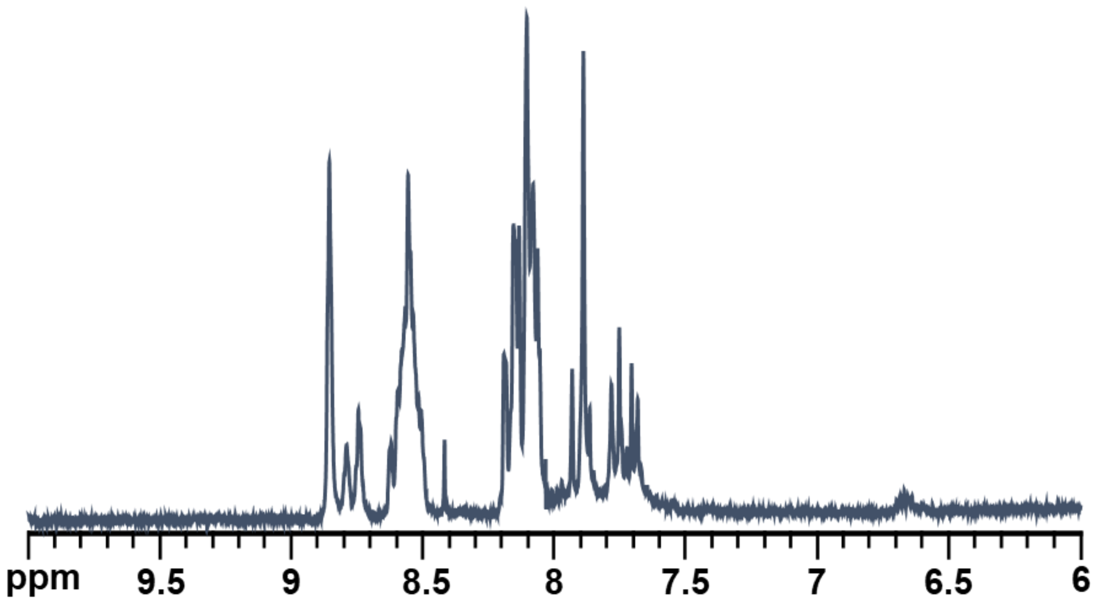

Figure S13. 1H NMR spectra of **3** in D_2O.

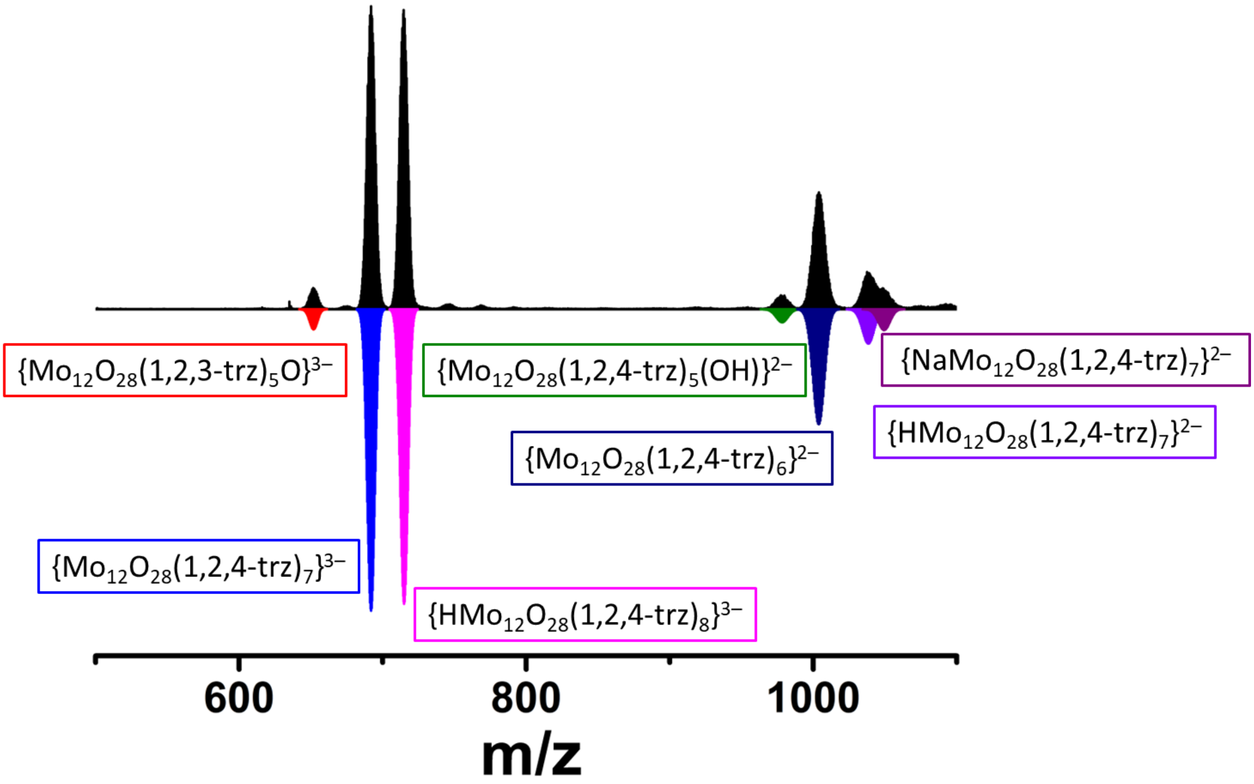

Figure S14. HR-ESI-MS spectrum of solution of **3** in water (black) and simulations of cluster forms (colored).

NMR-spectra of 2 after precipitation with HCl

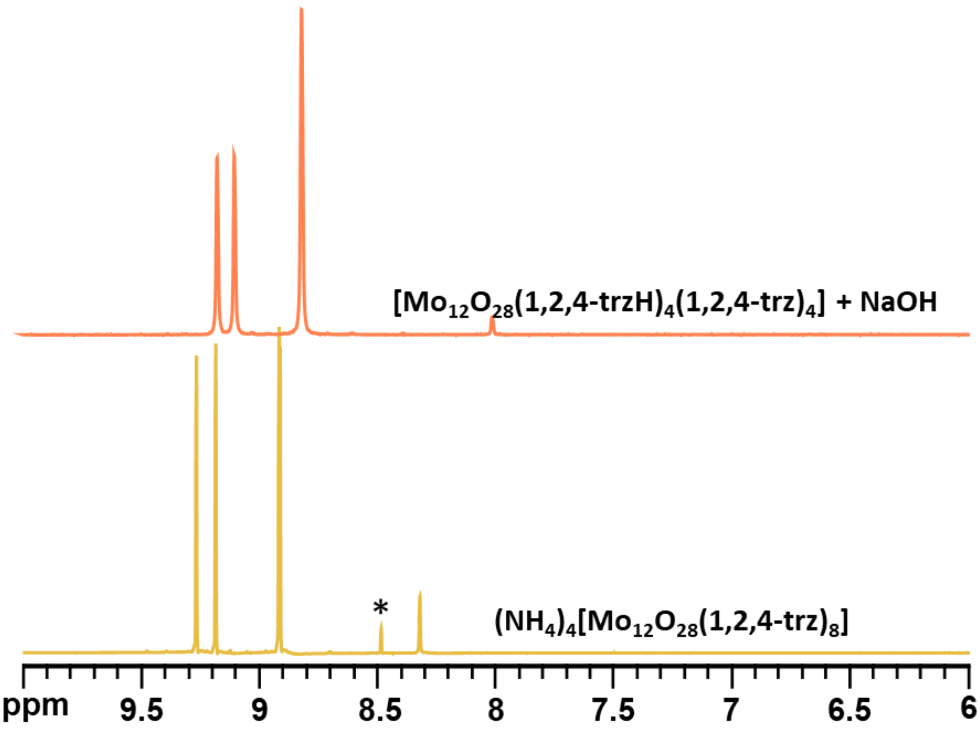

Figure S15. ^{1}H NMR spectra of **2** before (bottom) precipitation with HCl and spectra of dissolved precipitate with NaOH (top) in D_2O.

UV-vis spectra of solutions in culture medium

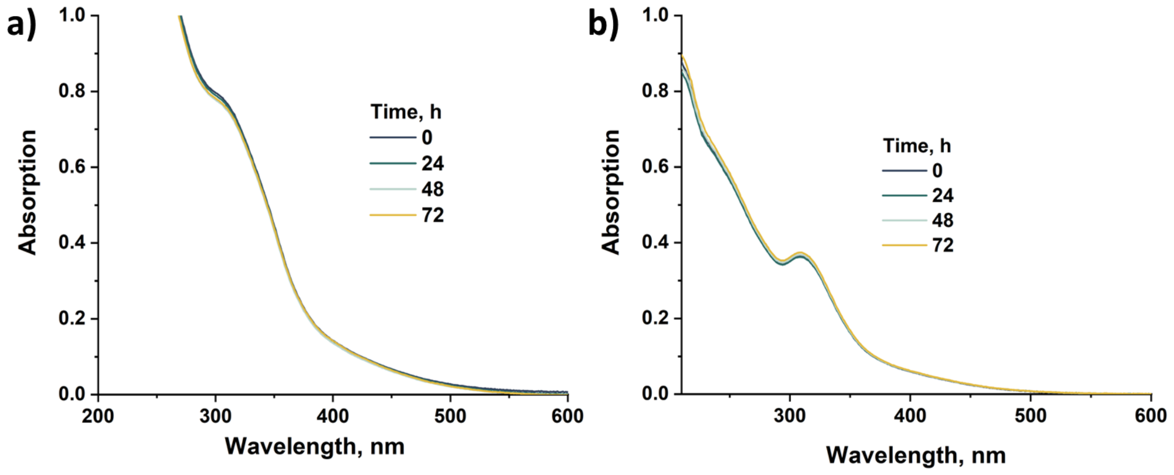

Figure S16. UV-vis spectra of compounds **1** (a) and **2** (b) in culture medium in time.

ICP-AES data

Table S2. Quantities of Mo accumulated by cells (Hep-2 and MRC-5) after incubation in 1 mM (∽2350 µg Mo / $5 \cdot 10^5$ cells) of solutions of **1** and **2** for 30 minutes, 2, 6, 24 and 48 h.

**Sample type**	**Time**	**Hep-2, µg Mo/$5 \cdot 10^5$ cells**	**MRC-5, µg Mo/$5 \cdot 10^5$ cells**
Control		0.02	0.01
**1**	30 minutes	0.25	0.22
	2 hours	0.46	0.25
	6 hours	0.47	0.24
	24 hours	0.75	0.77
	48 hours	0.75	0.83
**2**	30 minutes	0.18	0.23
	2 hours	0.32	0.11
	6 hours	0.33	0.12
	24 hours	0.43	0.34
	48 hours	0.42	0.34

IR spectra

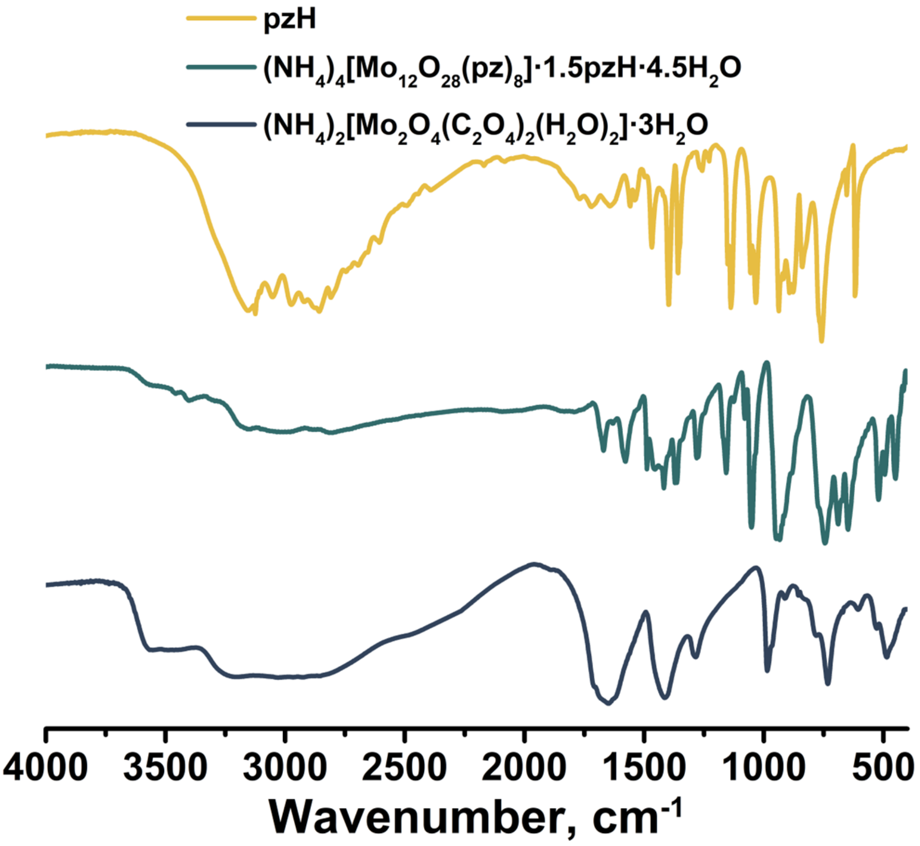

Figure S17. FTIR spectra of **1** in comparison with pyrazole and initial cluster.

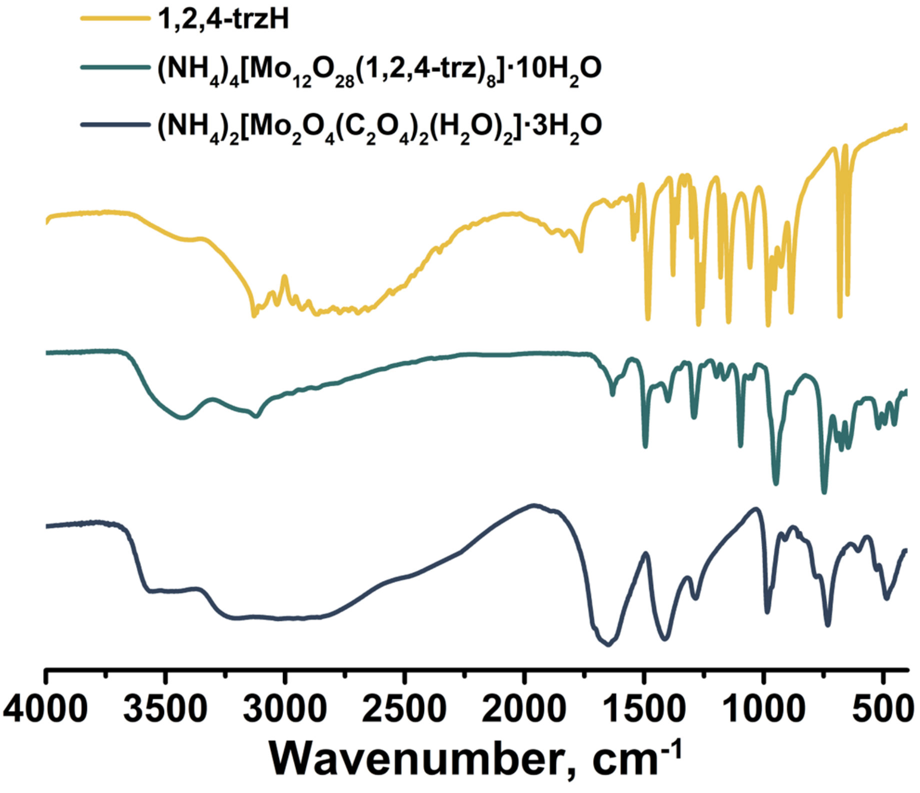

Figure S18. FTIR spectra of **2** in comparison with pyrazole and initial cluster.

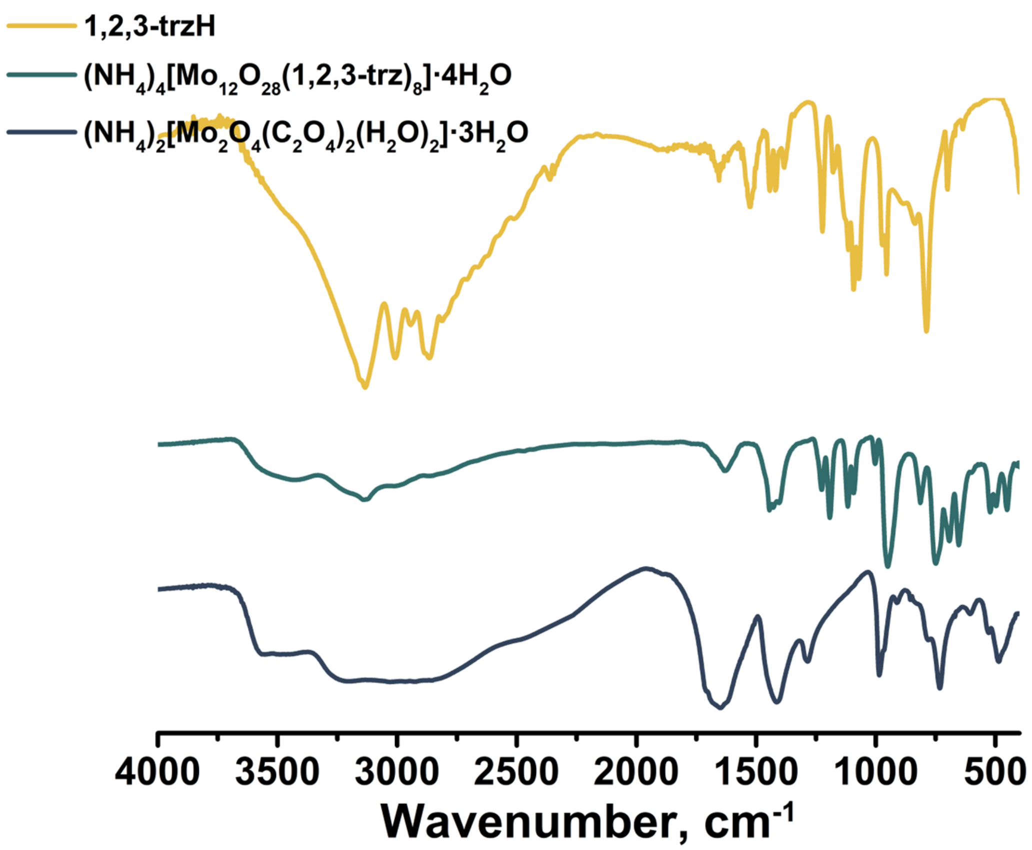

Figure S19. FTIR spectra of **3** in comparison with pyrazole and initial cluster.

Thermogravimetric analysis

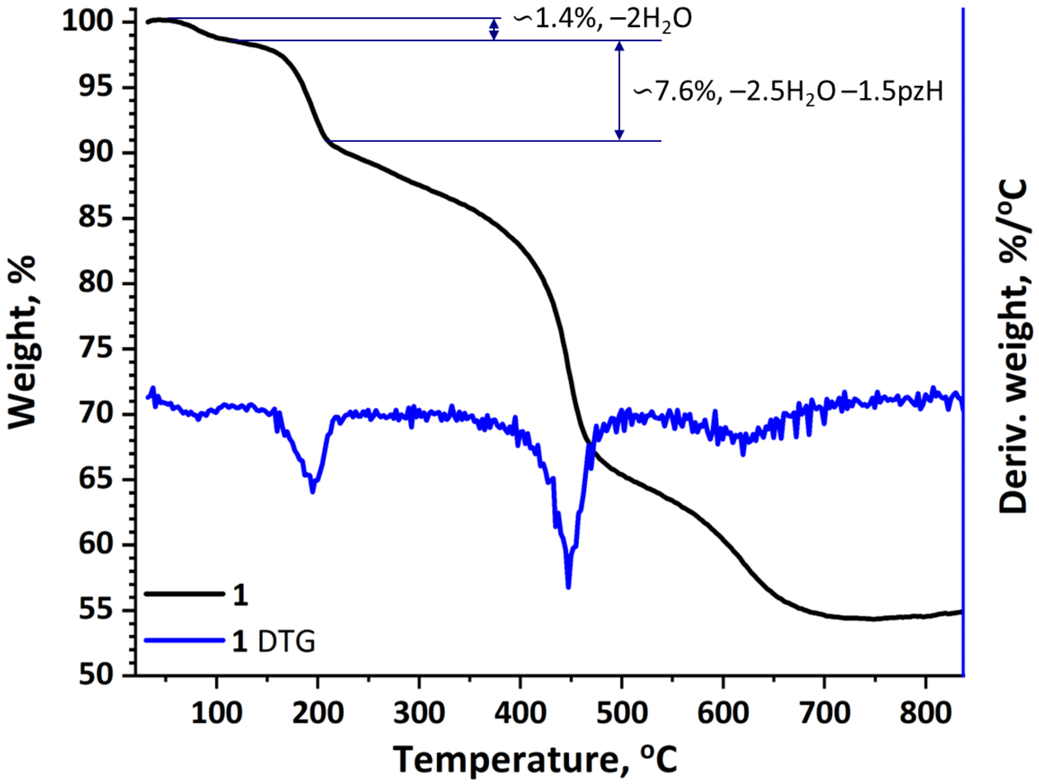

Figure S20. TGA and DTG curves of **1**. Heating rates of 10 $^{0}C{\cdot}min^{-1}$.

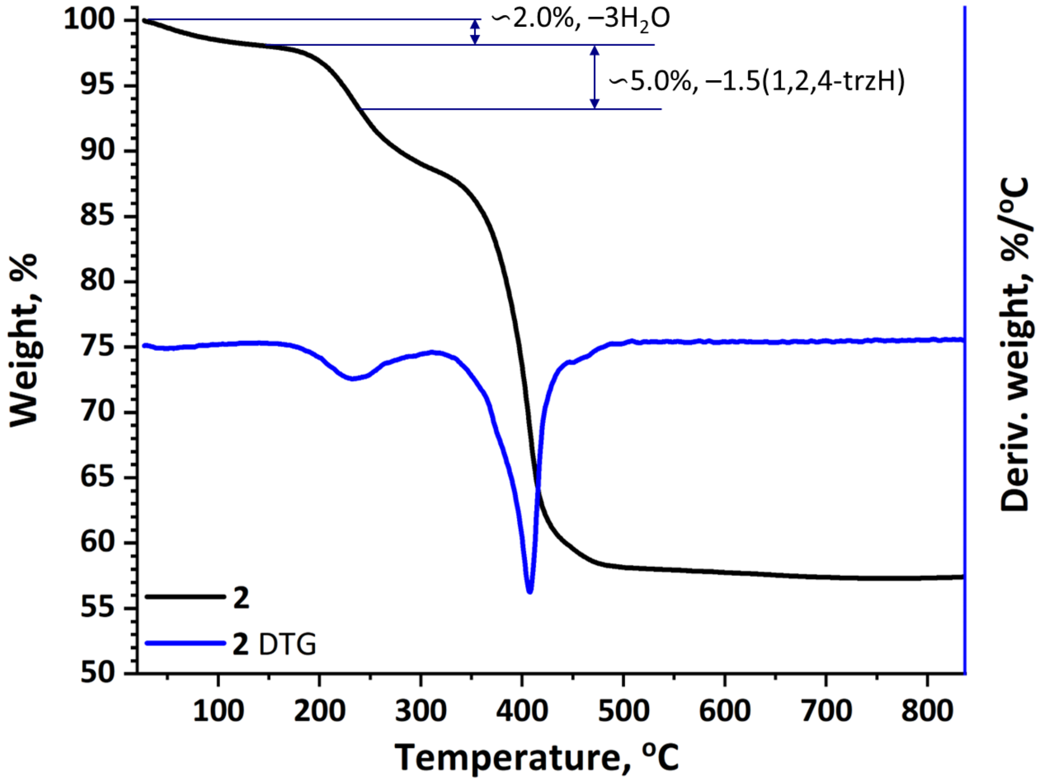

Figure S21. TGA and DTG curves of **2**. Heating rates of 10 $^{0}C{\cdot}min^{-1}$.

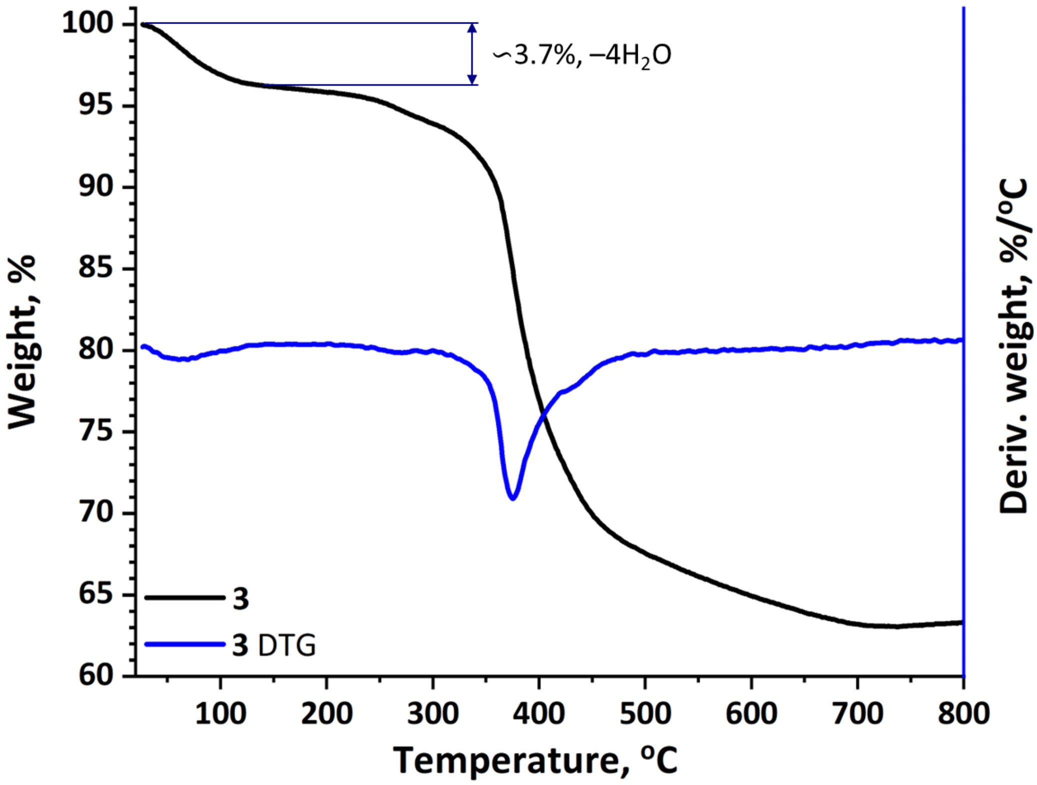

Figure S22. TGA and DTG curves of **3**. Heating rates of 10 $^{o}C{\cdot}min^{-1}$.

Powder X-ray diffraction analysis

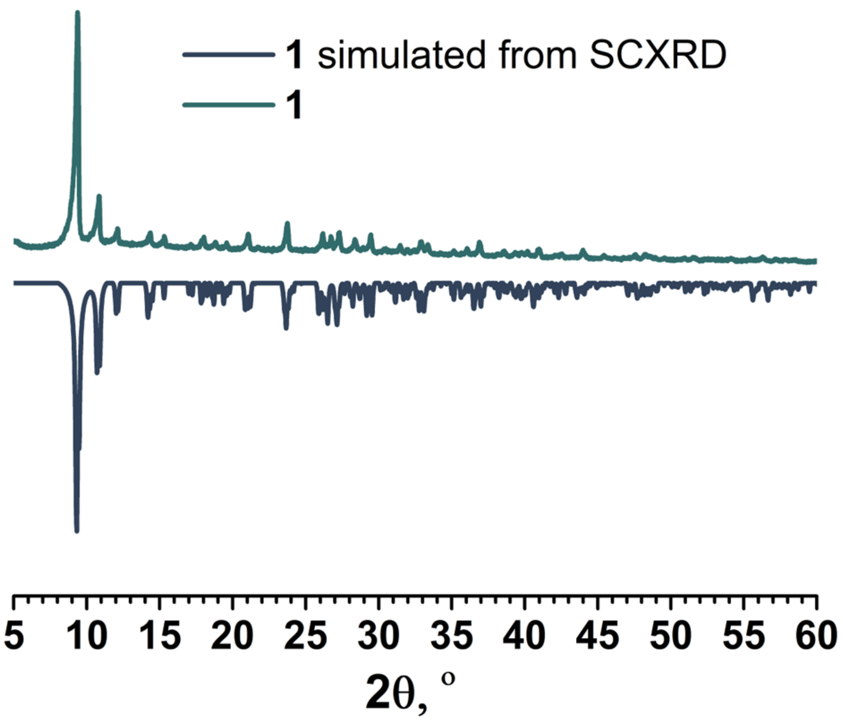

Figure S23. XRPD pattern of **1** in comparison with calculated one from the SCXRD data.

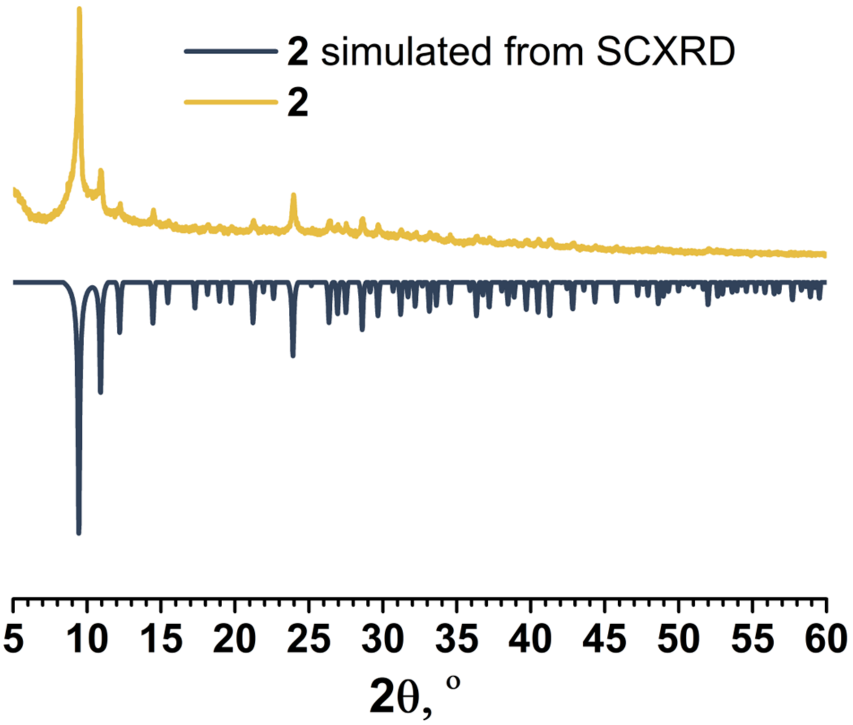

Figure S24. XRPD pattern of **2** in comparison with calculated one from the SCXRD data.

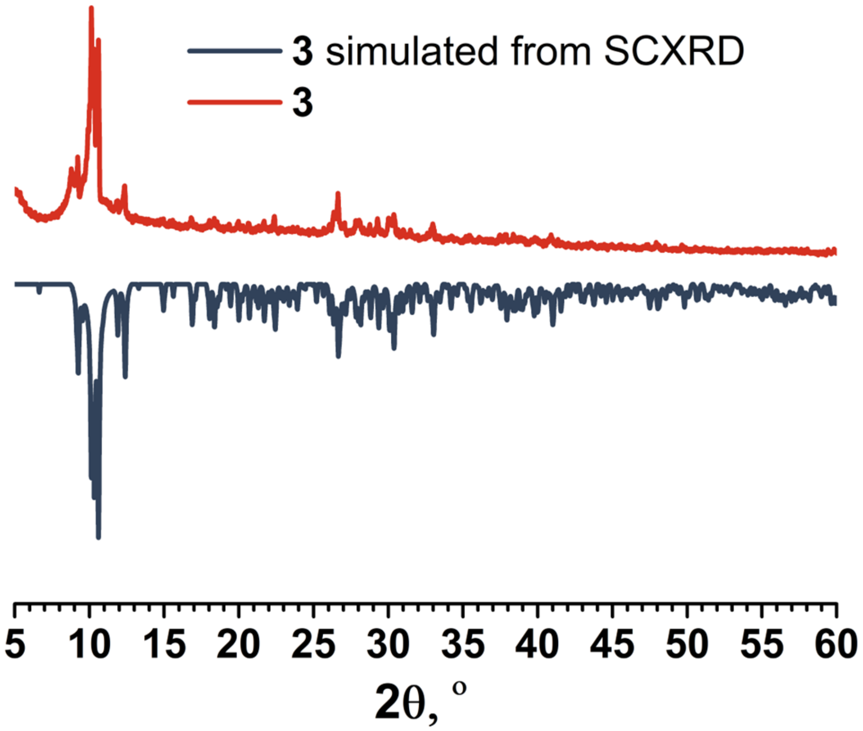

Figure S25. XRPD pattern of **3** in comparison with calculated one from the SCXRD data.

Crystal structure data

Table S3. Selected crystallographic parameters of the single-crystal X-ray diffraction structural analysis for $(NH_4)_4[Mo_{12}O_{28}(pz)_8]\cdot1.5pzH\cdot4.5H_2O$ (**1**), $(NH_4)_4[Mo_{12}O_{28}(1,2,4\text{-trz})_8]\cdot10H_2O$ (**2**) and $(NH_4)_4[Mo_{12}O_{28}(1,2,3\text{-trz})_8]\cdot4H_2O$ (**3**).

Compound	**1**	**2**	**3**
CCDC	2299185	2299187	2299186
Empirical formula	$C_{28.5}H_{55}Mo_{12}N_{23}O_{32.5}$	$C_{16}H_{52}Mo_{12}N_{28}O_{38}$	$C_{16}H_{40}Mo_{12}N_{28}O_{32}$
Formula weight	2391.23	2396.13	2288.04
Temperature, K	150(2)	150(2)	150(2)
Crystal system	Trigonal	Cubic	Monoclinic
Space group	*R* 3c	$I\ \bar{4}3d$	$P\ 2_1/n$
a, Å	38.3453(15)	26.6106(4)	11.7496(4)
b, Å	38.3453(15)	26.6106(4)	21.5118(7)
c, Å	22.8946(11)	26.6106(4)	22.6938(7)
α, °	90	90	90
β, °	90	90	102.1740(10)
γ, °	120	90	90
V, Å^3	29153(3)	18843.6(8)	5607.0(3)
Z	18	12	4
ρ_{calc}, g/cm^3	2.452	2.534	2.710
μ, mm^{-1}	2.338	2.420	2.696
F (000)	20718	13824	4368
Crystal size	0.12 × 0.10 × 0.10	0.06 × 0.06 × 0.06	0.10 × 0.08 × 0.08
2Θ range for data collection, °	1.850 to 33.172	2.165 to 30.495	1.893 to 33.158
Index ranges	$-58 \le h \le 58$<br>$-58 \le k \le 45$<br>$-35 \le l \le 35$	$-37 \le h \le 37$<br>$-38 \le k \le 37$<br>$-38 \le l \le 38$	$-18 \le h \le 18$<br>$-33 \le k \le 33$<br>$-28 \le l \le 34$
Reflections collected	184771	180189	74359
Independent reflections	24695 [R_{int} = 0.0853]	4789 [R_{int} = 0.0811]	21345 [R_{int} = 0.0520]
Data/restraints/parameters	24695/19/918	4789/24/254	21345/30/832
Goodness-of-fit on F^2	1.069	1.131	1.063
R_1 / wR_2(I > 2σ(I))	0.0514/0.1212	0.0243/0.0549	0.0385/0.0850
R_1 / wR_1 (all data)	0.0580/0.1249	0.0275/0.0565	0.0606/0.0914
$\Delta\rho_{max}/\Delta\rho_{min}$ (e·Å^{-3})	2.197/–2.089	0.640/–0.506	1.260/–1.537

References

1. M. K. Ehlert, S. J. Rettig, A. Storr, R. C. Thompson and J. Trotter, *Inorg. Chem.*, 1993, **32**, 5176–5182.
2. B. Modec, J. V. Brenčič, L. Golič and L. M. Daniels, *Polyhedron*, 2000, **19**, 1407-1414.
